# Pearl‐Like Bioinspired Coating Enables Regulation of Mg Degradation for Osteoporotic Bone Repair

**DOI:** 10.1002/advs.202521927

**Published:** 2025-12-20

**Authors:** Siming Zhang, Tao Zhang, Yuan Chen, Nikolaos Kourkoumelis, Mo Chen, Jiale Dong, Zhenyu Li, Yanling Zhou, Ning Li, Chen Zhu, Xifu Shang, Jiaxiang Bai, Xianzuo Zhang

**Affiliations:** ^1^ Department of Orthopedics, Centre for Leading Medicine and Advanced Technologies of IHM, The First Affiliated Hospital of USTC, Division of Life Sciences and Medicine University of Science and Technology of China Hefei Anhui China; ^2^ Department of Orthopaedics The Second Affiliated Hospital of Anhui University of Chinese Medicine Hefei Anhui China; ^3^ Department of Medical Physics University of Ioannina Ioannina Greece

**Keywords:** magnesium alloy, osseointegration, osteoporosis, pearl‐like structure

## Abstract

In osteoporotic bones, the stability of orthopedic implants is compromised, and excessive M1 macrophage polarization at the bone‐implant interface disrupts bone‐immune homeostasis, leading to implant loosening or failure. To address this, this study develops a bionic magnesium alloy internal fixation coating inspired by the “brick‐and‐mortar” structure of pearl, aiming to improve bone‐implant integration and vascularization in osteoporotic conditions. The multifunctional coating consists of a calcium phosphate (Ca‐P) “brick” layer, which serves as a mineralization template and corrosion barrier, and fibronectin‐mimetic peptides (Fn‐mimetic peptides) as the “mortar” to promote cell adhesion, regulate immune responses, and stimulate angiogenesis. This bionic multilayer structure not only alleviates oxidative stress in the osteoporotic microenvironment but also fosters immune regulation‐osteogenesis coupling and improves the bone‐vascular‐immune microenvironment. It precisely controls the degradation rate of Mg alloys and enhances tissue repair. The CaP layer reduces rapid degradation and prevents hydrogen gas release and local alkalinization, whereas Fn‐mimetic peptides enhance early bone integration and vascularization. The synergistic effect of the magnesium alloy implant and bionic coating significantly improved bone implant stability, regeneration, and vascularization, as demonstrated in osteoporotic rat models, offering a promising strategy for the design of bone repair materials under pathological conditions.

## Graphical abstract

In this study, **inspired by pearl architecture**, we developed a layered Ca–P “brick” and Fn‐mimetic peptide “mortar” coating on **Mg alloys**. The Ca–P layer moderates corrosion and offers mineral cues, while the peptide enhances adhesion and regulates immune‐vascular responses. This design aims to match Mg degradation with tissue repair, enabling faster, more stable osseointegration in osteoporotic bone.

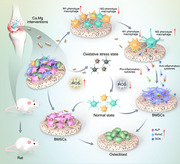

## 1 Introduction

Bone repair materials and internal fixation implants have immense clinical potential. However, existing metallic devices have notable limitations [1, 2]. Current standard implants are made of inert metals, such as titanium or tantalum alloys, which are mechanically robust and biologically inactive. These permanent metallic constructs often necessitate secondary surgical removal because of long‐term complications such as stress‐shielding‐induced bone resorption, aseptic loosening, and chronic inflammatory responses [3, 4]. Moreover, mechanical supports exhibit suboptimal bone healing and integration under pathological conditions with compromised osteogenic capacity, such as osteoporosis [5]. Inert metal implants provide structural support but cannot actively promote bone regeneration, leading to poor osseointegration, particularly in weakened or osteoporotic bones [6, 7].

Magnesium (Mg) and its alloys have emerged as promising alternatives for orthopedic implants [8, 9]. Their greatest advantage lies in their biodegradability: they gradually corrode in vivo into magnesium ions (Mg^2^⁺) and other harmless products, which can be safely metabolized by the body, thereby eliminating the need for secondary removal surgery [10, 11]. The released magnesium ions are not only biocompatible but also promote bone healing by modulating macrophages toward a pro‐osteogenic phenotype and activating angiogenic signaling. This process couples immune regulation with osteogenesis and remains effective even under pathological conditions such as osteoporosis [12, 13]. In addition, magnesium enhances local angiogenesis and alleviates oxidative stress, further optimizing the bone‐regeneration microenvironment. These attributes make magnesium alloys a highly bioactive platform for internal fixation materials [14].

Bare magnesium corrodes too rapidly in body fluids, producing hydrogen gas and increasing local pH. This disrupts early tissue contact and reduces mechanical support, especially in osteoporotic bone [15]. The theoretical gas yield is ∼1 L H_2_ per gram of Mg, so corrosion must be strictly controlled [16, 17, 18]. Excessively fast degradation can also cause the implant to lose mechanical support before the bone heals, leading to alkalization and interface disruption that hinder osseointegration. Osseointegration proceeds through three stages—inflammation, proliferation, and remodeling. Early on, it requires promoting the M1 to M2 macrophage shift, restraining oxidative stress, and activating angiogenesis. Materials that regulate these responses can improve bone–implant bonding in adverse conditions such as osteoporosis [16, 19, 20]. Thus, an ideal implant should balance mechanical matching, controlled degradation, and coordinated modulation of immune, antioxidant, angiogenic, and osteogenic processes.

Surface coatings satisfy these requirements. Calcium‐phosphate (Ca‐P) layers slow corrosion, buffer the interface, and provide an osteoconductive mineral template [21, 22, 23]. In nature, pearl achieves outstanding damage tolerance and interfacial energy dissipation through its “brick‐and‐mortar” lamellar structure, offering key inspiration for addressing the mechanical degradation and interfacial instability of Mg alloys in wet environments. Compared with conventional surface treatments, coatings that merely retard corrosion can no longer satisfy the demands of complex tissue repair; by contrast, bioinspired, pearl‐like brick‐and‐mortar architectures markedly improve coating stability and damage resistance in body fluids via crack deflection, crack bridging, and platelet pull‐out mechanisms, yielding high specific strength and toughness [24, 25]. Building on this, integrating cell‐adhesive motifs (e.g., fibronectin‐mimetic, RGD‐containing sequences) with functional Ca‐P layers into such hierarchical structures can strengthen integrin‐mediated adhesion and enhance in vivo bone–implant contact and early fixation [26, 27, 28]. More importantly, this synergistic “structure + biofunction” strategy not only controls corrosion and mechanical decay but also co‐regulates immune responses, restrains oxidative stress, promotes angiogenesis, and drives osteogenic differentiation. In this way, a more favorable and stable bone–implant interface can be established at an early stage while aligning Mg resorption with tissue repair. Likewise, magnesium‐rich or catalytic coatings can further modulate local biology by attenuating oxidative stress, stimulating angiogenesis, and coordinating immune responses to facilitate healing [29]. Overall, an optimally engineered, bioinspired functional coating can transform a magnesium implant from an inert support into an active therapeutic device that couples mechanical support with osteoinductive and immunomodulatory cues [30].

**SCHEME 1:**
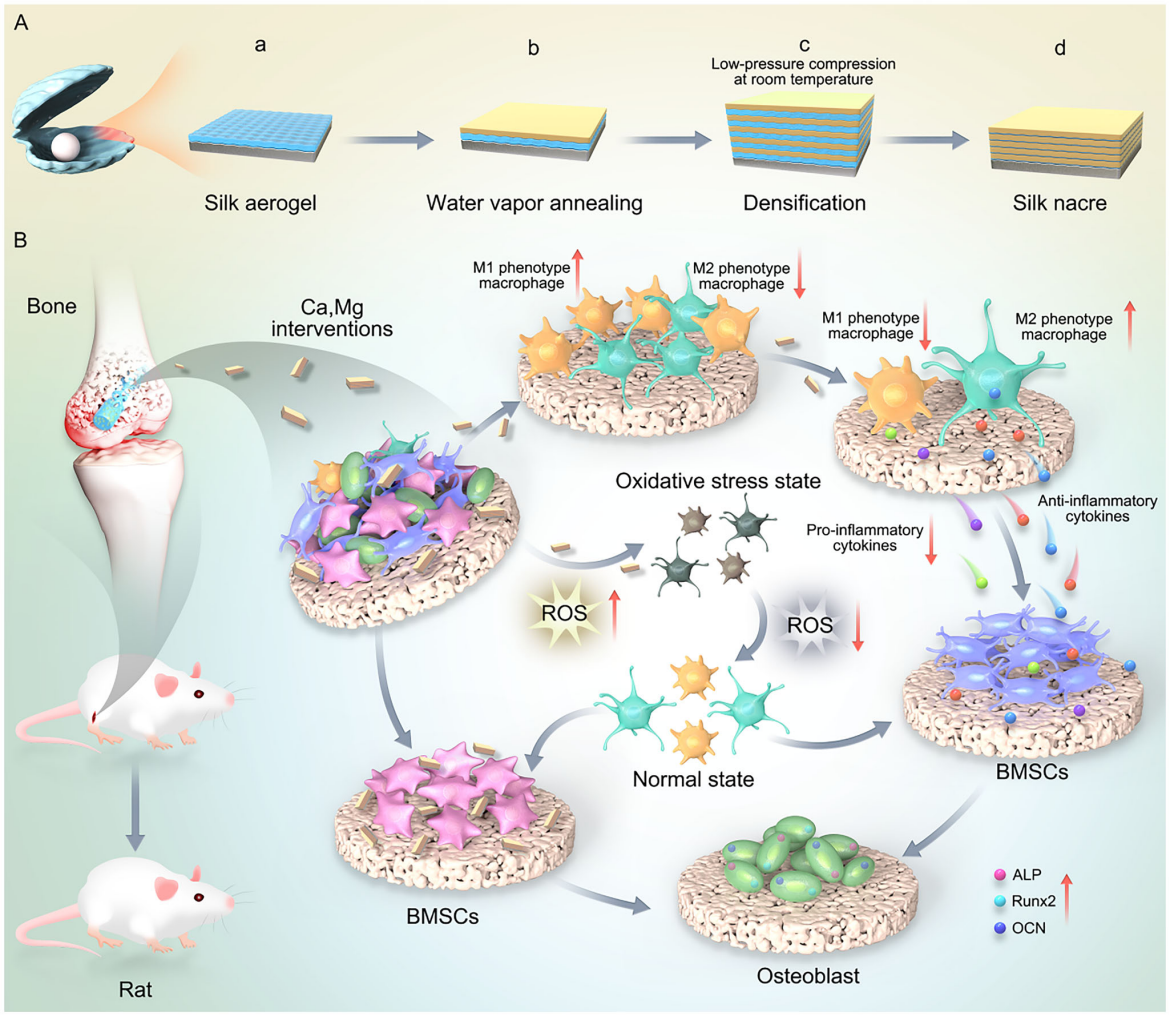
Schematic of the synthesis of the Fn‐modified pearl‐like structure coating and its biomedical applications.

In this study, we engineer a pearl‐inspired Fn‐mimetic peptide + Ca‐P layered coating on Mg alloy. Ca‐P “bricks” moderate corrosion and supply mineral cues. An Fn‐mimetic “mortar” promotes cell adhesion and supports immune and vascular balance. The layered design targets kinetic matching between Mg resorption and tissue repair, aiming for faster, more stable osseointegration in osteoporotic bone. (Scheme 1). Overall, the layered coating strategy, featuring a pearl‐like structure that integrates Ca‐P mineralization with bioactive peptides, offers an innovative solution for the application of Mg alloys in bone repair. This approach demonstrates significant potential for achieving rapid and stable bone integration, particularly in addressing clinical challenges, such as osteoporosis.

## 2 Result

### 2.1 Surface Modification and Characterization

**FIGURE 1:**
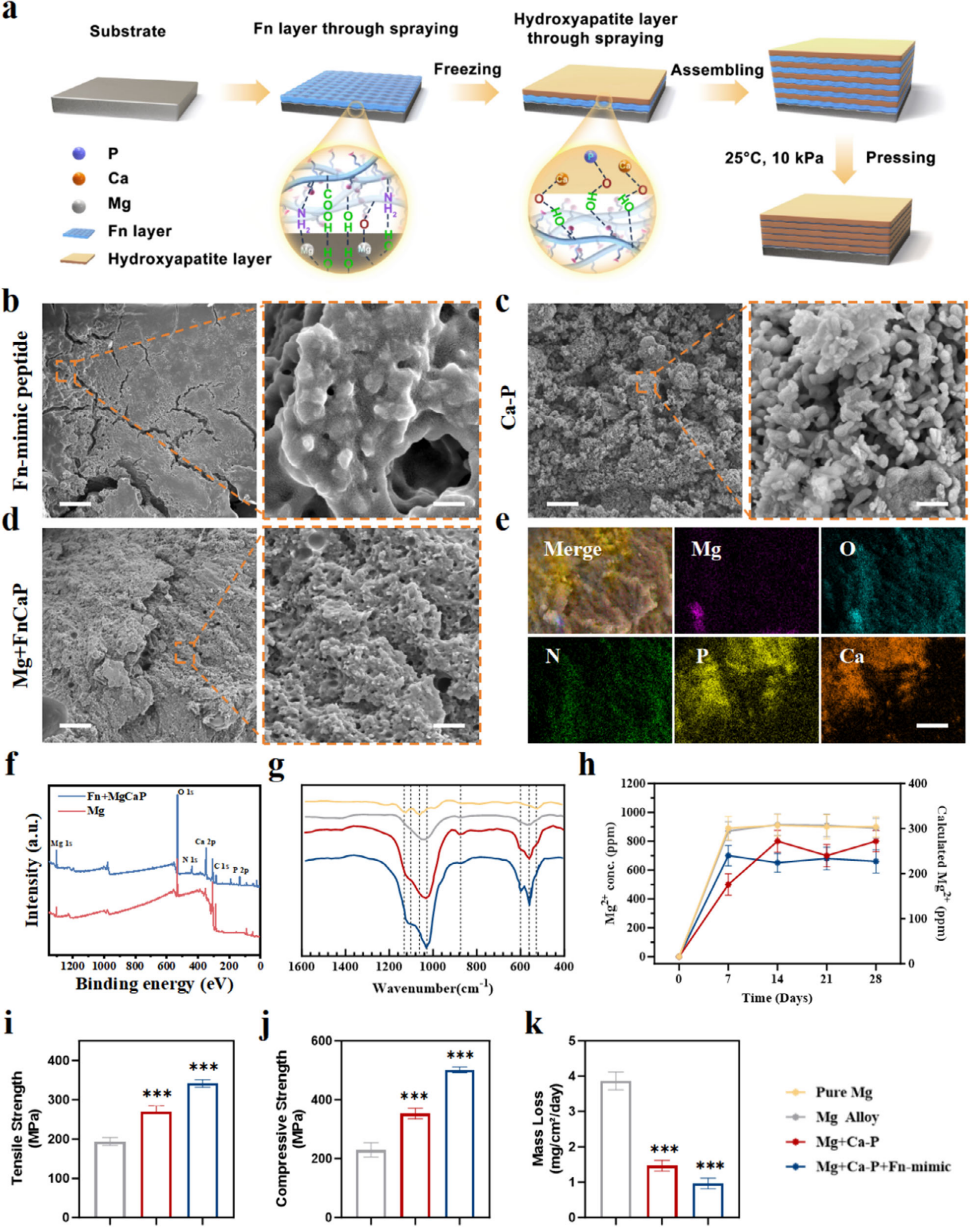
Preparation and characterization of Mg+FnCaP. a) Schematic of Mg+FnCaP synthesis. b–d) Representative SEM images of Fn‐mimic peptide, CaP and Mg+FnCaP, scale bar = 5 µm (left), and 200 µm (right) (n = 3). e) EDS element mapping of Mg+FnCaP, scale bar = 200 µm. f) XPS patterns of the bare and modified Mg surfaces (Mg and Mg+FnCaP) (n = 3). g) FTIR Analysis of Bare and Modified Mg Surfaces: Pure Mg, Mg Alloy, Mg+CaP, and Mg+FnCaP (n = 3). h) ICP Analysis of Mg^2+^ from Different Groups: Pure Mg, Mg Alloy, Mg+CaP, and Mg+FnCaP (n = 3). i) Tensile Strength of Mg Alloy, Mg+CaP, and Mg+FnCaP (n = 3). j) Compressive Strength of Mg Alloy, Mg+CaP, and Mg+FnCaP (n = 3). k) Mass loss per unit area of Mg Alloy, Mg+CaP, and Mg+FnCaP (n = 3). Data shown represent the mean ± SD. Statistical analysis was performed using one‐way ANOVA with a Tukey's post hoc test. Compared with Control, *p < 0.05, ***p < 0.001.

The surface morphology, composition, and interfacial structure of the Mg alloy matrix and coatings were studied using various characterization methods. Figure 1a shows a schematic diagram of the material synthesis process. The surface morphologies of the corresponding synthesized materials (Magnesium alloy substrate, Fn peptide coating, and Ca‐P coating) are shown in the SEM images in Figure S1 and Figure 1b, c, respectively. Figure S1 shows that the surface of Mg was relatively smooth. Figure 1b shows the Fn‐mimetic peptide coating, which formed a dense, continuous protein film on its surface. A fine fiber network was intertwined to form an irregular mesh structure. Localized areas exhibited slight ridges due to protein molecule folding or aggregation, and the edges showed layered peeling of the film. The overall morphology resembled the Fn layer in the natural extracellular matrix, demonstrating good structural integrity and a certain degree of flexibility. Figure 1c shows the SEM morphology of hydroxyapatite, which exhibits uniformly distributed spherical or layered microcrystalline structures with 3D surface porosity, mimicking the morphology of natural bone minerals. Microcrystals aggregate into clusters, thereby increasing their surface area, which is crucial for biomedical applications. Additionally, the 3D network structure of hydroxyapatite not only enhances the mechanical interlocking between the coating and calcium phosphate “bricks” but also provides abundant chemical bonding sites, significantly improving the overall stability and functional performance of the coating. Figure 1d shows the typical SEM characteristics of the “pearl‐like structure Fn‐coated magnesium alloy.” In this structure, calcium phosphate particles (‘bricks’) are uniformly distributed and embedded in a fibrous network matrix formed by a coating of fibrous simulated protein peptides (Fn, “mud”). Through elemental mapping analysis (Figure 1e), the distributions of Mg, O, N, P, and Ca were further verified, clearly revealing the doping characteristics of high calcium and phosphorus content in the final magnesium alloy. The network matrix covers the surface of the magnesium oxide (MgO) skeleton, forming a tightly bonded interface. The Fn‐like coating effectively filled the interstitial spaces between the particles and interacted with the calcium phosphate particles and MgO framework, providing a stable bonding strength. Additionally, the coating surface exhibited moderate roughness and micron‐scale pore structures, which enhanced the functional performance of the material. Further verification of the composition of the modified material was conducted via X‐ray photoelectron spectroscopy (XPS) (Figure 1f), confirming the presence of calcium phosphate particles and magnesium oxide frameworks and revealing the chemical bonding relationships between them. The XPS spectrum of the Mg+FnCaP coating (blue) showed intense O 1s, Ca 2p, P 2p, and N 1s peaks, whereas the Mg 1s signal from the substrate was markedly attenuated. These results indicate that the Mg alloy surface was effectively shielded by the Fn‐mimetic peptide/Ca–P multilayer. The weak residual Mg signal may have originated from minor substrate contributions through the thinner regions of the coating. EDS elemental mapping consistently showed that Mg was only locally distributed (Figure 1e), in contrast to the uniform distribution of Ca, P, O, and N, confirming that the Mg substrate was effectively covered and that the coating formed a continuous, pearl‐inspired organic–inorganic hybrid layer.

This provides strong evidence supporting the “pearl‐like structure Fn‐like coating” morphology observed via SEM. The longitudinal SEM (Figure S2) of the “pearl‐like Fn‐coated magnesium alloy” shows a layered structure with a clear hierarchy and smooth transition, and the layers are tightly bonded without obvious cracks or defects, which demonstrates excellent structural integrity. By integrating the mechanical support of the MgO skeleton, the bioactivity of calcium‐phosphorus particles, and the immunomodulatory and cell adhesion properties of Fn, this pearl‐like layered structure offers significant improvements. This enhances the mechanical strength, interfacial stability, and osseointegration of the material. This enhancement provides a strong foundation for future research.

We obtained the open‐circuit potential (OCP)/time curve, potential/logarithmic current density (applied voltage/logarithmic current density (A/cm^2^)), polarization curves, real and imaginary components of impedance (Zre and Zimg) in the Nyquist plot, and the frequency‐impedance modulus (frequency/Z) in the Bode plot. The results (Figure S3) showed that both the fibrous polypeptide‐calcium phosphate (FN‐p/Ca‐P) and calcium phosphate (Ca‐P) coatings significantly shifted the corrosion potential to a more positive value, reduced the corrosion current density, and improved the charge‐transfer resistance and low‐frequency impedance modulus. These enhancements in electrochemical parameters demonstrate that both coatings exhibit excellent corrosion resistance and superior interface stability, providing strong support for the use of Mg alloys in orthopedic implant applications. The chemical composition of the Mg alloy coating was subsequently analyzed using Fourier transform infrared spectroscopy (FTIR) (Figure 1g). Bulk Mg and Mg alloys are essentially featureless in the mid‐IR range, and weak features arise from the native surface film and minor atmospheric or handling artifacts, such as MgO/Mg(OH)_2_ lattice/OH modes, carbonate/water, or polishing residues. Traces of adsorbed phosphate can also appear as weak shoulders near 1050 cm^−1^ (ν3PO_4_^3−^), sometimes with features around 560–600 cm^−1^ (ν_4_). The strong absorption contour at 950–1150 cm^−1^ is attributed to the ν_1_ and ν_3_ normal modes of the apatitic phosphate ion. Mg+CaP (red) exhibits the phosphate ν_3_ band envelope at ≈1000–1100 cm^−1^ together with a broadened ν_4_ region near ≈600 and ≈560 cm^−1^ as well as a band at approximately 870 cm^−1^ corresponding to the ν_2_ symmetric bending mode of CO_3_^2−^ ions. These features are consistent with a carbonated, poorly crystalline calcium phosphate phase. In contrast, Mg+FnCaP (blue) displayed a narrower ν_3_ envelope and a well‐resolved ν_4_PO_4_^3−^ doublet (≈600/560 cm^−1^), indicative of more apatitic crystallinity; the ν_1_ mode at ≈960 cm^−1^ was partially resolved as a weak shoulder, suggesting the apatite lattice's non‐ideal local symmetry of the PO_4_ tetrahedron. Overall, the Mg+FnCaP spectra indicated a higher apatitic order and crystallinity, which is consistent with larger and/or better‐ordered domains. They also confirm the formation of the pearl‐inspired organic–inorganic hybrid and show that the Fn‐mimetic peptide enhances mineral order relative to the Ca‐P coating, evidenced by a sharper ν_3_ envelope and a more clearly split ν_4_ doublet. Thus, the FTIR experiments suggest that Fn‐like sequences act as nanoscale templates that promote the nucleation and oriented growth of apatite platelets while preserving the pearl‐style layered architecture.

Finally, the release of Mg ions from the Mg alloy in simulated body fluids was tested using inductively coupled plasma emission spectroscopy (ICP‐OES) (Figure 1h). ICP‐OES measurements showed a rapid increase in dissolved Mg^2^⁺ during the first week for all groups, followed by near‐steady plateaus. Mg and the bare Mg alloy accumulated the highest Mg^2^⁺ levels (≈0.90 g·L^−1^), whereas the Ca‐P coating reduced ion release (≈0.75–0.80 g·L^−1^). The Mg+FnCaP multilayer further suppressed Mg^2^⁺ accumulation to ≈0.65 g·L^−1^ over 28 days, indicating superior degradation control. Therefore, the peptide layer adds continuity and seals defects beyond the Ca‐P coating alone, enabling controlled slow release of magnesium ions and creating a long‐lasting, bone‐supportive environment for tissue repair. These findings further confirmed the protective effect of the coating on the substrate and its biofunctionality on the matrix.

Based on the above analyses of structural and surface characteristics, the experiments further validated the significant advantages of the Fn‐type pearlite structure coating on Mg alloys, particularly in terms of practical performance. First, in terms of the tensile strength (Figure 1i), the coating effectively enhanced the load‐bearing capacity of the material under tensile loading, enabling it to adapt to complex mechanical environments. In terms of the yield strength (Figure 1j), the coating significantly expanded the elastic deformation range of the material and increased the critical stress level, thereby ensuring the safety and deformation resistance of the material during use. Additionally, in terms of water contact angle (Figure S4), the coating significantly reduced the material's contact angle, indicating enhanced hydrophilicity. This change not only improves the wettability and surface cleanliness of the material but also improves the compatibility of the coating with other materials or media, thereby enhancing the adaptability and functionality of the material under various environmental conditions. Furthermore, in terms of degradation performance (Figure 1k), the coating effectively slowed down mass loss per unit area of the material, improving its stability and durability in long‐term use. This ensures that the material maintains an excellent performance under environmental influences and extends its service life. Specifically, the Fn‐type coating with a pearlite structure significantly reduces the degradation rate of the magnesium alloy, thereby achieving a dynamic match with the bone regeneration rate. Its degradation products uniformly release bioactive ions, further promoting bone tissue repair. These comprehensive performance improvements indicate that the Fn‐like‐coated Mg alloy is a reliable material for bone repair.

### 2.2 In Vitro Cytotoxicity Evaluation

Osteoporotic fractures are difficult to heal, and their pathological microenvironment imposes extremely high demands on the biocompatibility of implants [31]. Magnesium alloys are promising materials; however, their rapid degradation in fragile bone tissues compromises their cellular compatibility. Optimizing their degradation behavior and cellular response through surface coating is the key to enhancing their biocompatibility for osteoporosis applications [32]. This study focused on the need for osteoporosis repair by systematically evaluating the cellular compatibility of coated magnesium alloy materials through standard live/dead cell staining experiments (calcein‐AM/PI) and Cell Counting Kit‐8 (CCK‐8) cell proliferation experiments. Live/dead cell staining results (Figures S5, S6) revealed via fluorescence microscopy that, compared to the uncoated group, RAW264.7 and Bone Marrow Stem Cells (BMSCs) adhering to the coating‐modified group exhibited more extensive spreading morphology at both 24H and 72H, with fully extended cytoskeletons and well‐developed pseudopodia. The coated‐modified group exhibited a significant increase in live cells (green fluorescent labeling) with a dense distribution, whereas the proportion of dead cells (red fluorescent labeling) remained extremely low. These morphological observations and quantitative statistical results (Figure S7) collectively indicate that the coated‐modified Mg alloy provides a more suitable cell growth interface, with its excellent biocompatibility, effectively maintaining cell viability and laying a solid foundation for subsequent cell proliferation. The CCK‐8 assay results (Figure S8) showed that the coated magnesium alloy material effectively promoted the proliferation of cultured BMSCs and mouse macrophages (RAW264.7). At multiple time points (1 and 3days), the cells exhibited active growth and sustained proliferation trends, with no significant cytotoxic reactions observed. This indicated that the coating effectively buffered the adverse effects of the degradation products, providing support for cells in harsh environments. Based on these results, the coated Mg alloy material significantly promoted cell adhesion and proliferation without significant immunotoxicity, providing important theoretical and experimental evidence for its clinical application.

### 2.3 Immune Response Regulation Of Macrophage Polarization Phenotype by Pearl‐Like Coated Magnesium Alloy

Macrophages play a key role as effector cells in the osteoporosis immune microenvironment, profoundly influencing the homeostasis of bone metabolism [33, 34]. They are highly plastic and can polarize into both pro‐inflammatory M1 and anti‐inflammatory M2 subtypes in response to microenvironmental signals. In the immune microenvironment of osteoporosis, a significant increase in inflammatory factors (e.g., TNF‐α, IL‐1β, IL‐6, etc.) leads to an increase in the proportion of M1‐type macrophages and a decrease in the proportion of M2‐type macrophages, which, by secreting pro‐inflammatory factors, not only inhibit the proliferation and differentiation of osteoblasts, but also aggravate bone resorption through the activation of osteoclasts, which in turn disrupts the homeostasis of bone tissue. At the same time, M2‐type macrophages secreted fewer anti‐inflammatory factors and osteogenesis‐related growth factors, which significantly weakened bone regeneration and repair efficiency. This immune imbalance not only hinders bone tissue repair but also adversely affects the osseointegration of metal implants [35, 36]. Therefore, regulating macrophage polarization to restore immune homeostasis is important for promoting bone tissue repair and enhancing the osseointegration of metal implants.

**FIGURE 2:**
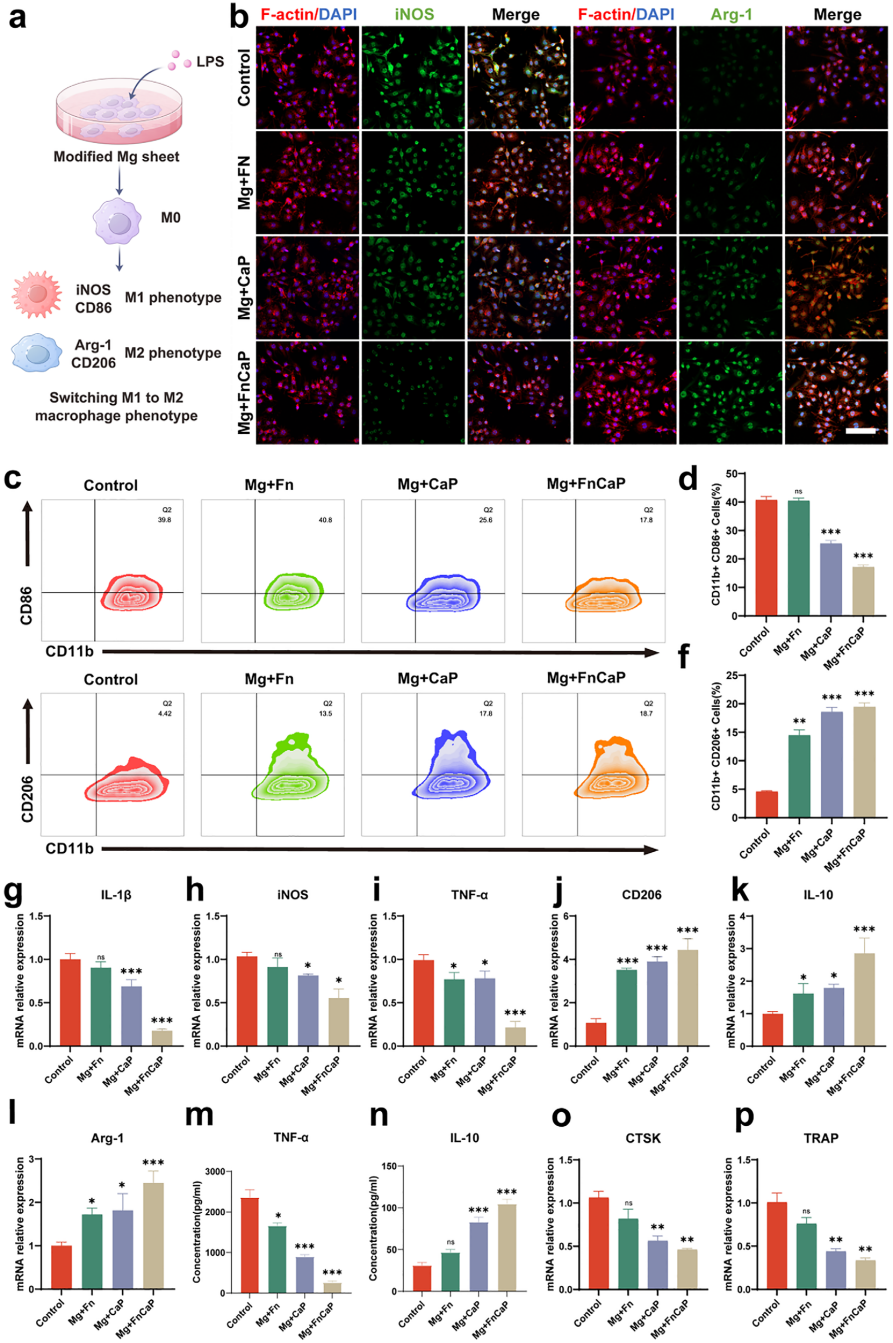
Immunomodulatory effects of Mg+FnCaP in vitro. a) Experimental design, b) Representative Confocal Laser Scanning Microscope(CLSM) images of RAW264.7 cells cultured on different samples (green: iNOS or Arg‐1; red: F‐actin; blue: nuclei). Scale bar = 50 µm (n = 3). c–f) Flow cytometry analysis of the expression of CD86 (M1) and CD206 (M2) in RAW264.7 cells cultured on different samples and quantitative results (n = 3), g–i) RT‐qPCR results of inflammatory cytokine genes (IL‐1β, iNOS, TNF‐α, CD206, IL‐10, and Arg‐1) expression in RAW264.7 cells (n = 3). m, n) Concentration of representative pro‐inflammatory cytokines in macrophage culture media after various treatments (n = 3). o, p) RT‒qPCR results of Osteoclast‐related gene (CTSK, TRAP) (n = 3). Data shown represent the mean ± SD. Statistical analysis was performed using one‐way ANOVA test with a Tukey's post hoc test. Compared with Control, *p < 0.05, **p < 0.01, ***p < 0.001, p > 0.05 were designated as not significant (ns).

To investigate the effect of different material matrices on macrophage polarization, we stimulated resting macrophage‐like RAW264.7 cells using lipopolysaccharide (LPS) to mimic the osteoporotic microenvironment and observed the phenotypic transformation (Figure 2a). To thoroughly assess the polarization state of macrophages, we employed immunofluorescence staining for iNOS and Arg‐1. As shown in Figure 2b and Figure S9 following LPS stimulation, the proportion of pro‐inflammatory M1‐type macrophages (iNOS+, green) significantly increased in the C and Mg + Fn groups, indicating that the macrophages polarized toward the pro‐inflammatory M1 type and participated in the local inflammatory response. In contrast, in the Mg+CaP and Mg + FnCaP groups, anti‐inflammatory M2‐type macrophages (Arg‐1+, green) were predominant, suggesting that these groups were more inclined toward anti‐inflammatory polarization, which helped suppress inflammation and promoted tissue repair. The flow cytometry (FCM) results also showed that after LPS stimulation, the expression ratio of the M1 surface marker CD86 was significantly increased in the C and Mg + Fn groups (Figure 2c, d), whereas the expression level of the M2 surface marker CD206 was markedly increased in the Mg+CaP and Mg + FnCaP groups (Figure 2e, f), further confirming the regulatory effect of the material combination on macrophage polarization. Additionally, quantitative RT‐qPCR analysis results showed that the expression of pro‐inflammatory M1 macrophage markers IL‐1β, iNOS, and TNF‐α was significantly upregulated (Figure 2g–i). In contrast, the expression of anti‐inflammatory M2 macrophage markers CD206, IL‐10, and Arg‐1 was significantly increased (Figure 2j–l), further confirming the significant advantage of the Mg and CaP material combination in promoting anti‐inflammatory immune responses. ELISA results further confirmed the above trend: TNF‐α release was significantly increased in groups C and Mg+Fn (Figure 2m), while IL‐10 release levels were significantly increased in groups Mg+CaP and Mg+FnCaP (Figure 2n), indicating that the material combination effectively induced M2 polarization and enhanced the anti‐inflammatory repair environment. It is worth noting that the PCR results also showed that the expression of the osteoclast‐related markers CTSK and (Tartrate‐Resistant Acid Phosphatase Staining) TRAP was significantly suppressed (Figure 2o, p), suggesting that the material combination not only promoted osteogenesis but also inhibited osteoclast activity, thereby contributing to the maintenance and regeneration of bone tissue homeostasis. These results comprehensively demonstrate that the combination of Mg and CaP materials can multidimensionally regulate the immune microenvironment, promote bone repair, and enhance the long‐term stability of implants.

### 2.4 High‐Throughput Sequencing Validates the Immunoregulatory Mechanism of Pearl‐Like Coated Magnesium Alloy

**FIGURE 3:**
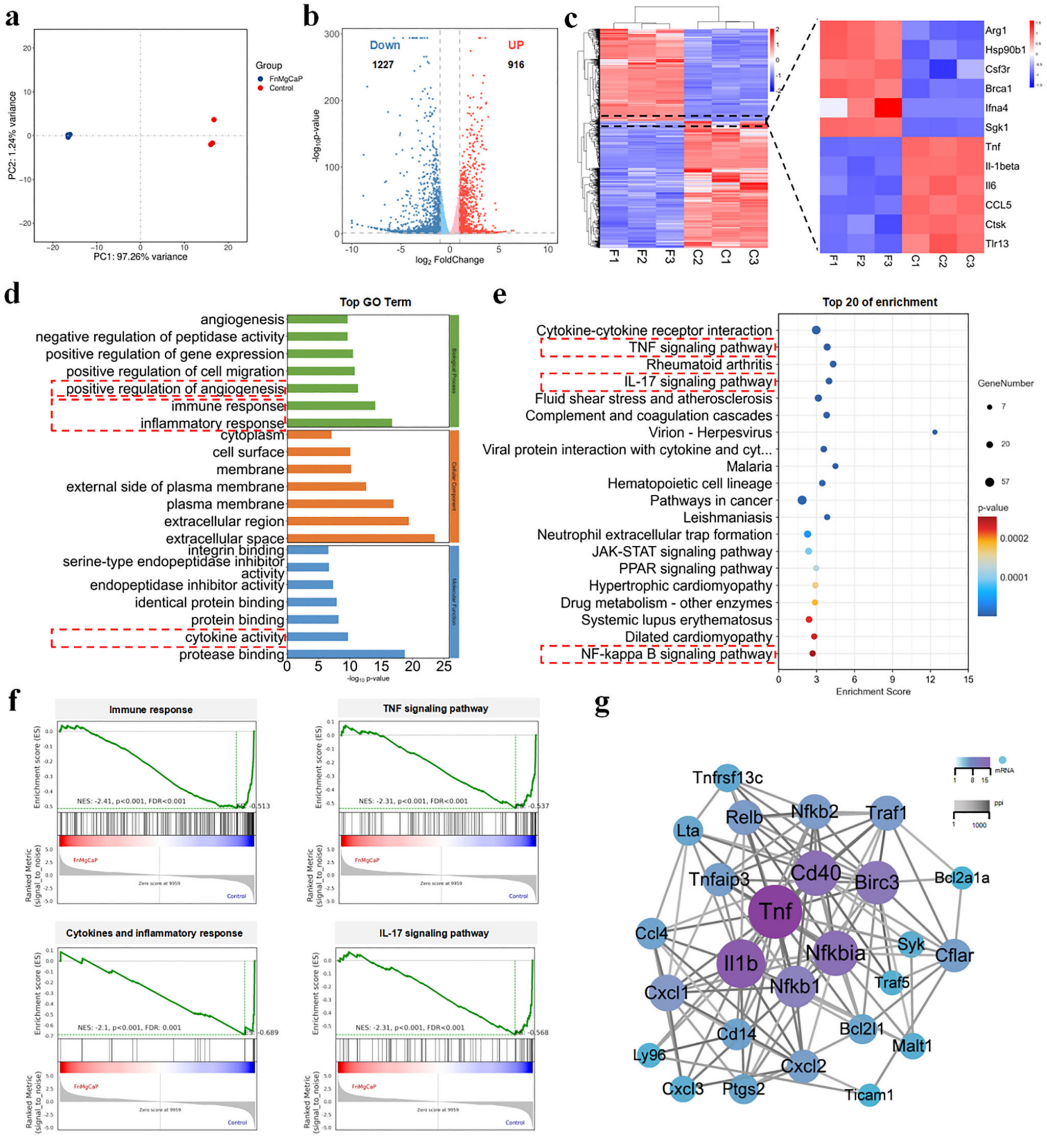
Transcriptomic Analysis of the Mechanisms by Which Mg+FnCaP Regulates Macrophage Polarization. a) PCA analysis of DEGs in Control and Mg+FnCaP samples (n = 3). b) Volcano plots of DEGs (n = 3). c) Heatmap of DEGs (n = 3). d) GO enrichment analysis of DEGs in macrophages cultured on Control *vs* Mg+FnCaP (n = 3). e) KEGG enrichment of downregulated genes (n = 3). f) Gene set enrichment analysis (GSEA) of the Immune response, Cytokines and inflammatory response, TNF, and IL‐17 signaling pathways(n = 3). g) Gene‐gene interaction network analysis of DEGs in the NF‐κB signaling pathway (n = 3).

In the above section, a pearl‐like‐coated magnesium alloy (Mg+FnCaP) demonstrated significant regulatory capabilities for gene expression in inflammation‐related environments associated with osteoporosis, particularly under pathological conditions characterized by heightened inflammatory responses. This finding prompted us to investigate further the potential mechanisms triggered by Mg+FnCaP‐treated macrophages and the biological events that they mediate. To elucidate the associated immunomodulatory mechanisms, RNA‐seq transcriptomic analysis was performed on macrophages cultured on the Mg alloys and Mg+FnCaP substrates. Principal component analysis (PCA) revealed a distinct separation between the treatment and control groups, with high reproducibility within each group, indicating robust data reliability (Figure 3a; Figure S10). Thousands of differentially expressed genes (DEGs) were identified in both the osteoporosis model and Mg+FnCaP‐treated groups. Volcano plot analysis revealed that compared to the osteoporosis group, 1,227 genes were significantly upregulated and 916 genes were significantly downregulated in the Mg+FnCaP group (Figure 3b). Statistically significant upregulated and downregulated DEGs are presented in Figure 3c, highlighting key genes associated with macrophage phenotype conversion and inflammatory factor expression. Mg+FnCaP treatment significantly suppressed LPS‐induced M1 macrophage activation, with marked downregulation of typical pro‐inflammatory factors TNF, IL‐1β, IL‐6, and chemokine CCL5, indicating its potent anti‐inflammatory effects. Concurrently, the M2 macrophage marker gene Arg‐1 was upregulated, further supporting the role of Mg + FnCaP in promoting anti‐inflammatory phenotype polarization. Additionally, Mg+FnCaP treatment led to a significant downregulation of CTSK, a key gene closely associated with osteoclast formation, suggesting its ability to effectively inhibit osteoclastogenesis and bone resorption processes. Notably, the antioxidant‐related genes Brca1 and Sgk1 were significantly upregulated in the treatment group, demonstrating the enhanced cellular antioxidant defense capabilities of Mg + FnCaP. In summary, Mg+FnCaP exhibited synergistic biological effects across multiple levels: it not only modulated macrophage immune phenotypes and suppressed inflammatory pathway activation, but also inhibited osteoclast formation and enhanced cellular antioxidant capacity. This study establishes a robust foundation for improving the inflammatory microenvironment associated with osteoporosis and promoting bone integration.

To further explore functional differences between the treatment and control groups in biological processes (BP), cellular components (CC), and molecular functions (MF), Gene Ontology (GO) enrichment analysis was performed. As shown in Figure 3d, multiple immune‐related biological processes were significantly enriched, primarily immune system processes, innate immune responses, inflammatory responses, angiogenesis, and cytokine activity (red‐boxed regions). These findings suggest that a pearl‐like‐coated magnesium alloy (Mg+FnCaP) may exert anti‐inflammatory effects by negatively regulating inflammatory responses and influencing the production of related inflammatory mediators. To further elucidate the potential signaling pathway mechanisms, RNA sequencing data were subjected to an in‐depth Kyoto Encyclopedia of Genes and Genomes (KEGG) enrichment analysis. Analysis revealed significant enrichment in multiple inflammation‐ and immunity‐related signaling pathways, including the IL‐17 signaling pathway, TNF signaling pathway, and downstream NF‐κB signaling pathway (Figure 3e). Notably, a higher proportion of genes were downregulated within these pro‐inflammatory pathways, suggesting that Mg+FnCaP effectively alleviated inflammatory responses by negatively regulating multiple pro‐inflammatory pathways. Subsequently, all the downregulated differentially expressed genes were subjected to GO, KEGG, and gene set enrichment analyses (GSEA). A comprehensive analysis further validated the pivotal role of Mg + FnCaP in the negative regulation of immune responses, IL‐17 receptor signaling pathways, TNF signaling pathways, and other immune‐related processes (Figure 3f). Finally, the protein‐protein interaction (PPI) network constructed from differentially expressed genes revealed significant downregulation of multiple key inflammatory factor signaling pathways (e.g., TNF, IL‐1β, and IL‐6) (Figure 3g). Subsequently, we further validated the sequencing results through PCR. PCR results showed that the C group (LPS‐treated group) significantly downregulated the expression of the NF‐κB inhibitor IKBα and mildly upregulated the classical NF‐κB subunit p50. Additionally, LPS treatment significantly induced the expression of its downstream target genes TNF‐α and IL‐1β, indicating that the NF‐κB pathway was activated. Mg+FnCaP intervention effectively reversed these changes, restored IKBα expression, and significantly reduced the levels of p50, TNF‐α, and IL‐1β, presenting a typical “LPS activates NF‐κB, and the drug shuts it down” anti‐inflammatory trend (Figure S11). Collectively, these transcriptomic features indicate that Mg+FnCaP treatment effectively mitigated the harmful proinflammatory microenvironment induced by osteoporosis, establishing a favorable immunoregulatory foundation for subsequent osseointegration.

### 2.5 Pearl‐Like Coated Magnesium Alloy Enhances Antioxidant Activity

High levels of oxidative stress are prominent features of the microenvironment of osteoporosis [37]. Oxidative stress is caused by an imbalance between free radicals and antioxidant substances in the body, leading to excessive reactive oxygen species (ROS) production [38]. The excessive accumulation of ROS causes damage to bone cells (e.g., osteoblasts, osteoclasts) and affects normal bone metabolism [39, 40]. Oxidative stress not only directly damages osteoblasts but also contributes to the chronic inflammatory response to osteoporosis through the activation of inflammatory pathways (e.g., NF‐κB pathway), which further deteriorates the bone condition [41]. This sustained high oxidative stress and inflammation levels work together to maintain the microenvironment of osteoporosis in an unfavorable state, preventing effective restoration of bone density. Therefore, the need for antioxidants is becoming increasingly apparent. Neutralizing excess free radicals in the body using antioxidants can reduce oxidative stress, inhibit inflammatory responses, protect bone cells, and promote normal bone metabolism, thereby alleviating the pathology of osteoporosis and improving bone health [42].

**FIGURE 4:**
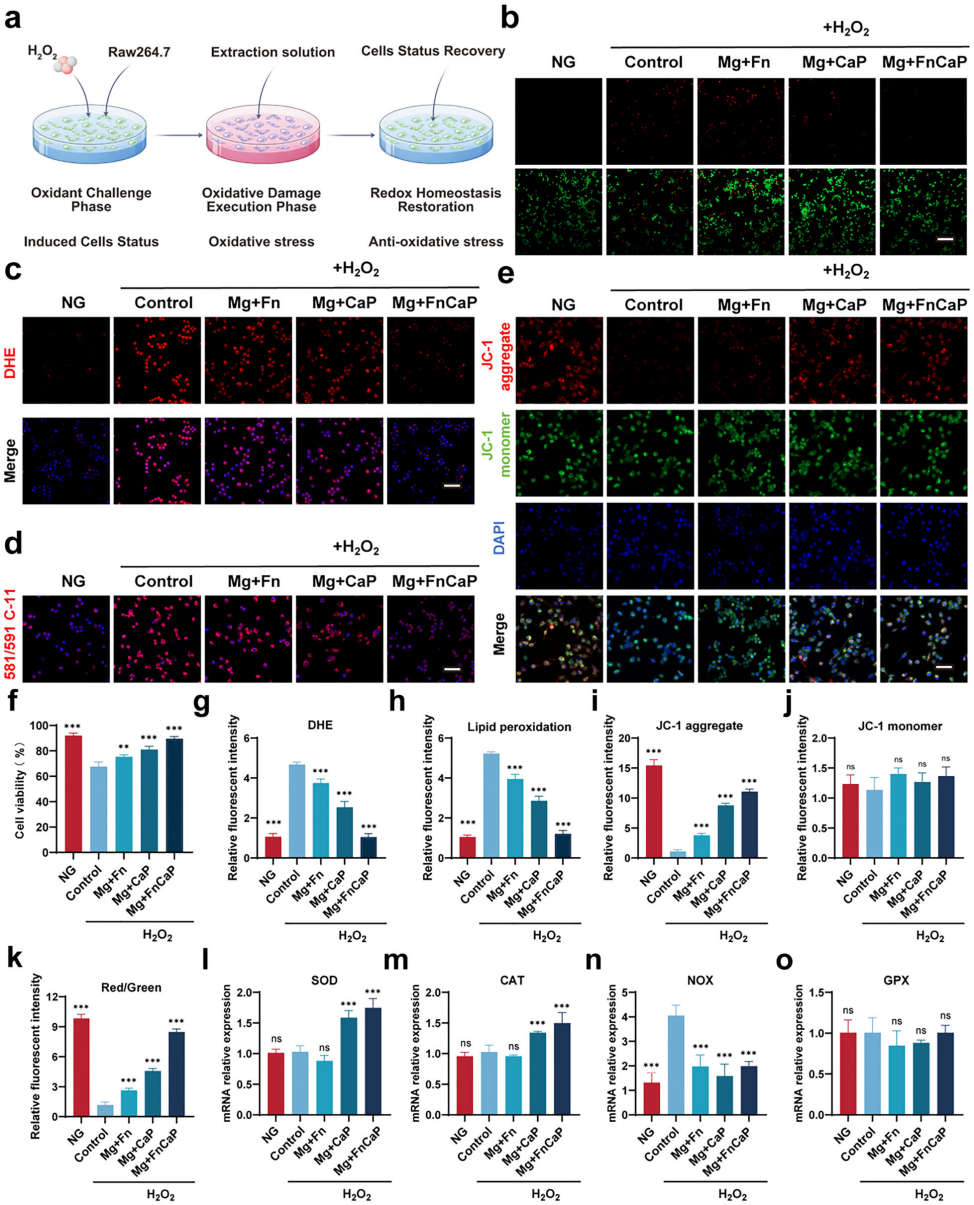
Antioxidant effects of Mg+FnCaP in vitro. a) Experimental design. b) Live/dead assay of RAW264.7 cells cultured on different surfacesand Scale bar = 25 µm (n = 3). f) quantitative results (n = 3). c) Immunofluorescence staining images of RAW264.7 cells (red: DHE; and blue: nuclei), Scale bar = 50 µm (n = 3) and g) quantitative results (n = 3). d) Representative fluorescence images of lipid peroxidation in macrophages after various treatments (n = 3). h) quantitative results, Scale bar = 50 µm (n = 3). e) Mitochondrial membrane potential (Δψm) levels were measured with the JC‐1 probe in RAW264.7 cells following treatment with different samples, Red and green fluorescence (n = 3) and i, j) quantitative results respectively (n = 3), correspond to JC‐1 aggregates and monomers, with Δψm corresponding to the ratio of red to green fluorescenceand k) quantitative results, Scale bar = 50 µm (n = 3). l–o) RT‒qPCR results of oxidative and antioxidant genes (SOD, CAT, NOX and GPX) expression in RAW264.7 cells (n = 3). Data shown represent the mean ± SD. Statistical analysis was performed using one‐way ANOVA test with a Tukey's post hoc test. Compared with Control, ns = no statistical significance,**p < 0.01, ***p < 0.001, p > 0.05 were designated as not significant (ns).

In osteoporosis, various cells, particularly macrophages, are chronically exposed to oxidative stress, making them vulnerable to damage by reactive oxygen species and other harmful factors. This leads to functional impairment, which, in turn, exacerbates the onset and progression of osteoporosis (Figure 4a). To simulate a microenvironment with high oxidative stress, cells were treated with hydrogen peroxide (H_2_O_2_) to treat the cells. To facilitate the comparison of oxidative stress effects on cell survival, the untreated cells were designated as the NG group, and the cells treated with H_2_O_2_ were designated as the Control group. The experimental results showed that under hydrogen peroxide induction, the number of cell deaths significantly increased, and cell survival rates markedly decreased. These results were further validated using live/dead cell staining, which revealed a marked increase in the number of dead cells. However, after adding different materials, the cell survival rates improved significantly, particularly in the Mg+FnCaP material group, where the number of dead cells was significantly reduced compared to that in the control group (Figure 4b, f). Subsequently, we evaluated the inhibitory effects of different materials on reactive oxygen species (ROS) accumulation using DHE staining. The results (Figure 4c, g) show that the Mg+FnCaP group exhibited significant ROS inhibitory effects under high oxidative stress conditions, significantly reducing ROS accumulation. Compared to the control group, the Mg+FnCaP group effectively alleviated the oxidative damage caused by hydrogen peroxide, further confirming its advantages in antioxidant therapy. The inhibitory effect of the materials on lipid peroxidation was evaluated using a paper‐based lipid peroxidation (LPO) assay. The results (Figure 4d, h) showed that the Mg+FnCaP group had significantly reduced LPO levels, with a marked reduction in lipid peroxidation compared to the control group, further demonstrating its excellent antioxidant effect. Finally, we used the JC‐1 reagent to detect mitochondrial membrane potential to assess the effects of each group of materials on mitochondrial function. The results (Figure 4e, i–k) showed that the Mg+FnCaP group significantly maintained the stability of the mitochondrial membrane potential and reduced the mitochondrial dysfunction caused by oxidative stress. Compared with the control group, the Mg+FnCaP group effectively protected the mitochondrial structure and function, further demonstrating its unique advantages in alleviating oxidative stress and promoting cellular health.

To further investigate the antioxidant mechanism, superoxide dismutase (SOD), catalase (CAT), nitric oxide synthase (NOX), and glutathione peroxidase (GPx) were selected as antioxidant enzyme markers. The results (Figure 4l–o) showed that the Mg+FnCaP group significantly enhanced the activity of SOD, CAT, and GPx, while reducing the activity of NOX, further enhancing the antioxidant capacity of the cells and effectively alleviating oxidative stress‐induced cellular damage. Compared to the control group, the Mg+FnCaP group demonstrated a significant advantage in regulating antioxidant enzyme activity, further confirming its role in alleviating oxidative damage. These findings provide mechanistic support for the antioxidant activity of this material and indicate that it effectively alleviates oxidative stress by enhancing the activity of endogenous antioxidant enzymes, thereby improving the osteoporotic microenvironment. This mechanism further revealed the potential of the Mg+FnCaP group in antioxidant therapy, providing a strong theoretical basis for its application in the treatment of osteoporosis.

### 2.6 Pearl‐Like Coated Magnesium Alloy Promotes Angiogenesis

Osteoporosis is significantly correlated with angiogenesis during bone repair [43, 44]. The formation of new blood vessels not only delivers oxygen, nutrients, and key growth factors (e.g., VEGF and BMPs) to osteoblasts but also provides microenvironmental support for bone matrix mineralization. However, in patients with severe osteoporosis, the bone marrow microvessel density is significantly reduced, suggesting that impaired angiogenesis may be an important driver of disease progression. It has been found that elevated pro‐inflammatory factors (e.g., IL‐6, TNF‐α) in the bone marrow microenvironment under osteoporosis pathology may impede neovascularization by inhibiting vascular endothelial cell function. At the same time, calcium ions and collagen degradation products released from overactive bone resorption not only damage the structural integrity of bone but also alter the stability of the vascular basement membrane, resulting in a vicious cycle of “vascular damage – bone metabolic imbalance” [45, 46]. This bidirectional regulation mechanism suggests a dynamic coupling between angiogenesis and bone remodeling, and targeting angiogenesis may provide a new direction for osteoporosis treatment, for example, by activating the HIF‐1α/VEGF pathway or by developing biomaterials with pro‐angiogenic properties.

**FIGURE 5:**
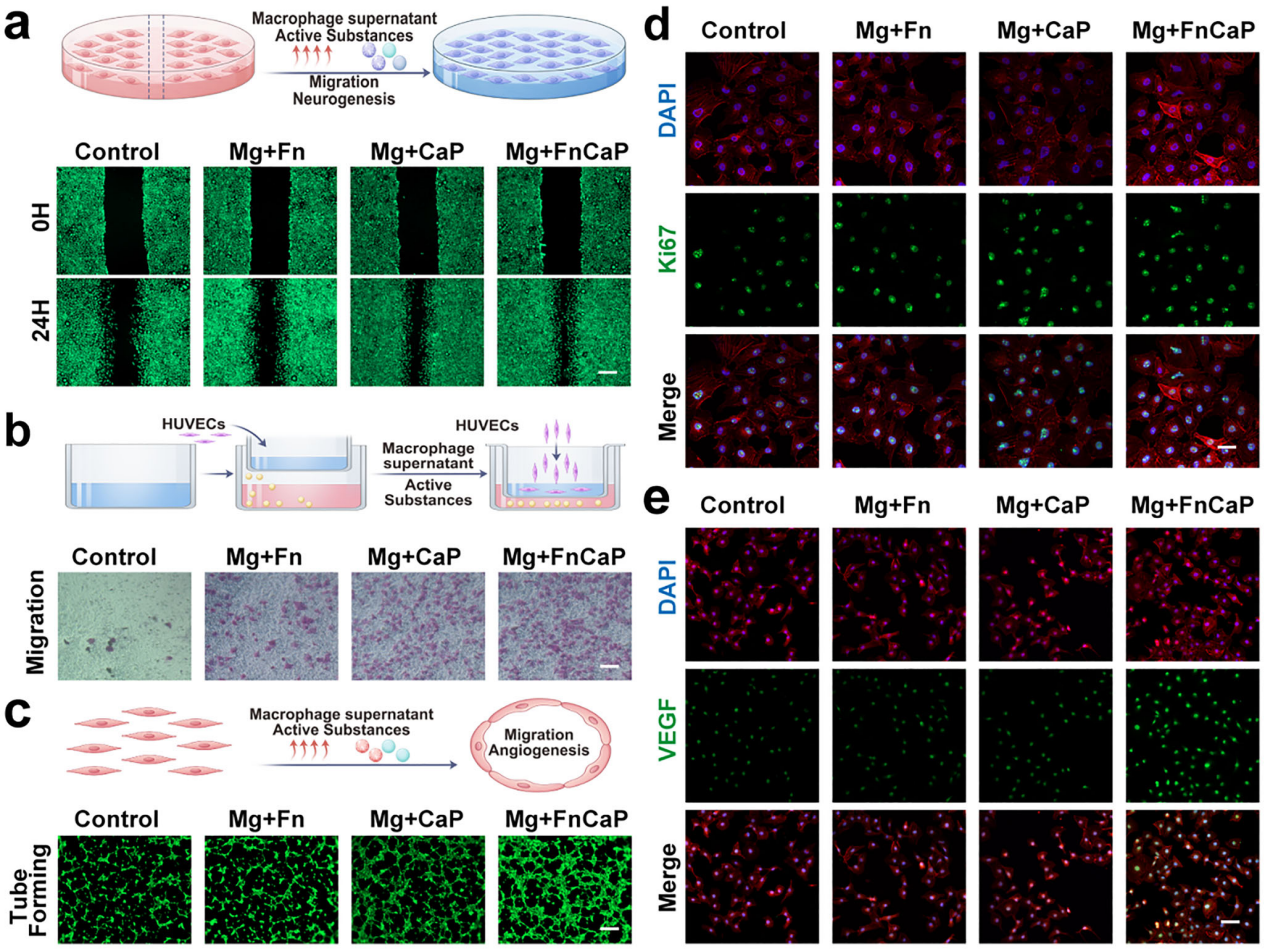
Effects of Mg+FnCaP on angiogenesis in vitro. a)Scratch wound healing tests assessed the effect of different samples on human umbilical vein endothelial cells (HUVECs) migration from 0 to 24 hours, Scale bar = 500 µm (n = 3). b) The effect of different samples on HUVECs migration was assessed by a 24‐hour Transwell method, Scale bar = 100 µm (n = 3). c) A 6‐hour tube formation assay assessed the ability of different samples to promote angiogenesis, Scale bar = 100 µm (n = 3). d, e) Immunofluorescence staining images of HUVECs (green: Ki67 or VEGF; red: F‐actin; blue: nuclei), scale bar = 50 µm (n = 3).

In this study, we used the HUVECs model to systematically evaluate the promotional effects of different implant materials on angiogenesis in three dimensions: cell migration, lumen formation, and molecular expression. Through various experimental methods, the potential of Mg+FnCaP the composite materials for in vitro angiogenesis was revealed. The scratch assay results (Figure 5a; Figure S12) showed that the wound healing rate in the Mg+Fn group was significantly higher than that in the control group, indicating that this material can significantly promote cell migration and wound repair. Within a 24‐hour observation period, the Mg+FnCaP group exhibited the best healing effect. This result was further validated by the transwell migration assay (Figure 5b; Figure S13), which demonstrated that the Mg+FnCaP group significantly enhanced the transcellular migratory ability of HUVECs. These results suggest that the Mg+FnCaP composite promotes the initial steps of angiogenesis by enhancing the migratory ability of endothelial cells. In the tubule formation experiment, the number of branching nodes formed by the Mg+FnCaP group within 6 h increased significantly (Figure 5c; Figure S14), and the complexity of the tubular network significantly improved. This experiment further demonstrated that the composite material promoted the formation of stable tubular structures by endothelial cells, thereby advancing angiogenesis. Additionally, the results of the Ki67/VEGF immunofluorescence double staining experiment (Figure 5d, e; Figure S15) showed that the fluorescence intensity in the Mg+FnCaP group increased in a dose‐dependent manner with the expression of vascular endothelial growth factors (such as Ki67 and VEGF), further validating that this material enhanced vascular endothelial growth through a dual mechanism of promoting endothelial cell proliferation and vascular endothelial growth factor secretion. This indicates that Mg+FnCaP composite materials not only promote endothelial cell proliferation, but also enhance the vascularization process by regulating the secretion of vascular endothelial growth factors, such as VEGF. Based on the above results, the Mg+FnCaP composite material demonstrated excellent pro‐angiogenic properties in an in vitro model. This finding provides new insights into overcoming treatment bottlenecks caused by limited angiogenesis in osteoporosis and offers important experimental evidence for the development of implant materials that combine bone integration and angiogenesis.

### 2.7 Pearl‐Like Coated Magnesium Alloy Enhances Osteogenic Differentiation

In the pathological microenvironment of osteoporosis, the proliferation, migration, and osteogenic differentiation capacity of BMSCs are significantly impaired, and this process involves multiple complex and intertwined factors [47, 48]. Osteoporosis is accompanied by a decrease in bone density and destruction of the bone microstructure, leading to significant changes in the physiological characteristics of the bone marrow microenvironment. These changes include reduced bone matrix mineralization, accumulation of harmful inflammatory factors, remodeling of the extracellular matrix, and damage to the bone marrow microvasculature [49]. Collectively, these factors disrupt the normal physiological functions of BMSCs. In this adverse microenvironment, the proliferative capacity of BMSCs is significantly reduced, cell cycle regulation is disrupted, osteogenic differentiation potential is limited, and their ability to migrate to sites of bone injury is greatly impaired. Furthermore, the pro‐inflammatory state and oxidative stress induced by osteoporosis further impair the reparative functions of BMSCs, limiting their role in bone reconstruction and repair. In summary, the complex microenvironmental changes induced by osteoporosis impair BMSCs function in multiple ways, thereby exacerbating the pathological progression of osteoporosis and significantly increasing treatment difficulties.

**FIGURE 6:**
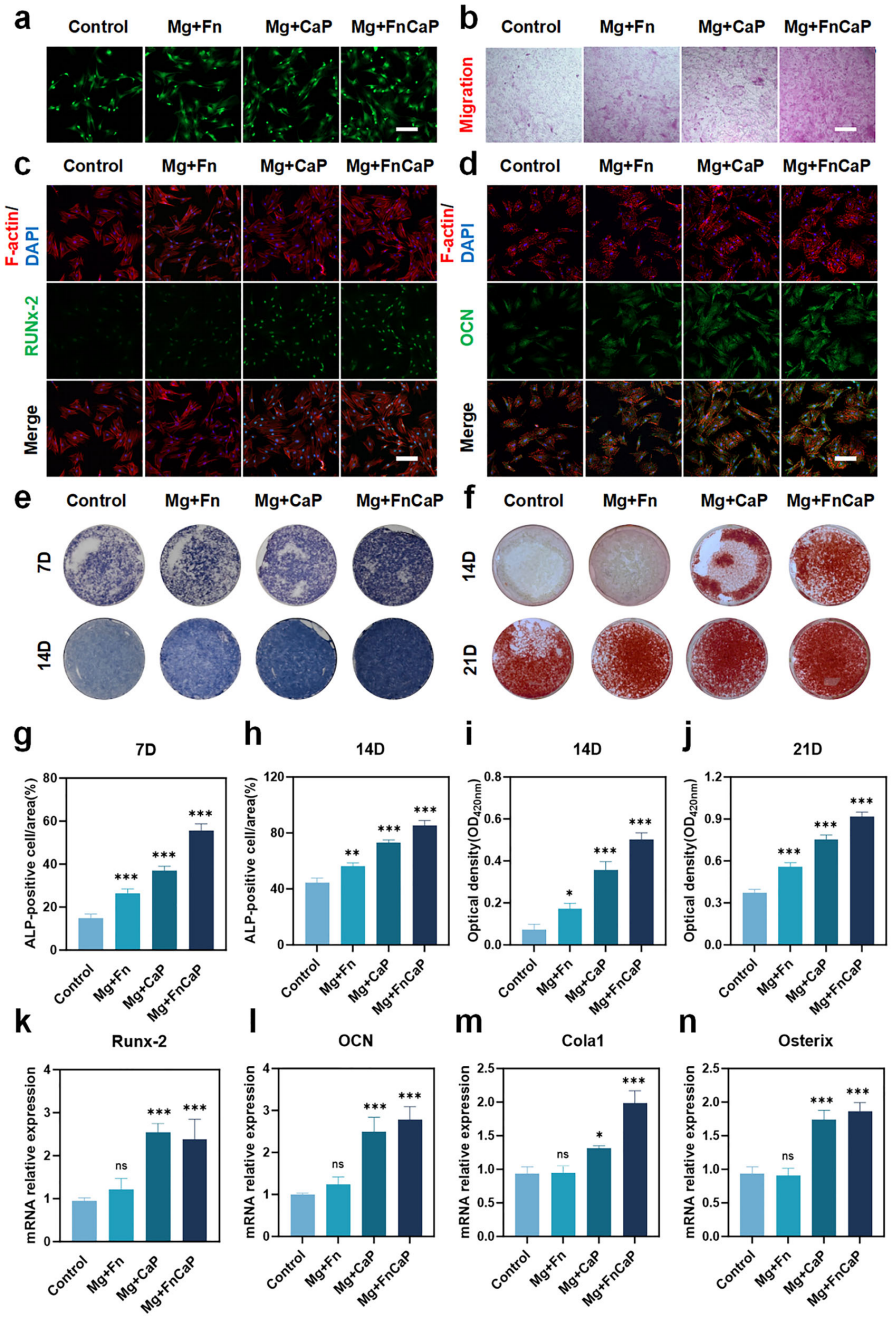
Osteogenic differentiation in vitro. a) Migration analysis of BMSCs after 4 hours of co‐culture with different group treatments, Scale bar = 50 µm (n = 3). b) The effect of different group on BMSCs migration was assessed by a 24‐hour Transwell method. Scale bar = 100 µm (n = 3). c, d) Immunofluorescent staining images of the BMSCs and mean fluorescence intensity (MFI) relative quantification (green: Runx2, OCN; red: cytoskeleton; blue: nuclei) (scar bar = 50 µm) (n = 3). e) ALP and f) ARS staining of BMSCs cultured in different conditioned medium and g–i) quantitative results, scale bar = 100 µm (n = 3). k–n) Osteogenesis‐related gene expression of BMSCs cultured in different conditioned medium (Runx2, OCN, Cola1, and Osterix) (n = 3). Data shown represent the mean ± SD. Statistical analysis was performed using one‐way ANOVA test with a Tukey's post hoc test. Compared with Control, *p < 0.05, **p < 0.01, ***p < 0.001, p > 0.05 were designated as not significant (ns).

To further investigate the effects of different magnesium alloy materials on the migration and differentiation of BMSCs, we cultured the isolated BMSCs on various magnesium alloy substrates (e.g., Mg group, Mg+Fn group, Mg+CaP group, and Mg+FnCaP group) and observed cell migration after 4 hours. The results (Figure 6a) show that the number of BMSCs significantly increased in all materials, particularly on the Mg+FnCaP alloy, where the number of BMSCs was notably higher than that in the other groups, indicating that this alloy has the most significant effect in promoting BMSCs migration. Transwell experimental results (Figure 6b; Figure S16) further confirmed that these materials effectively promoted BMSCs migration, with a gradually increasing trend in the Mg+Fn, Mg+CaP, and Mg+FnCaP groups, indicating that these materials significantly alleviated the inhibitory effect of the osteoporotic microenvironment on BMSCs migration. These results suggest that the Mg+FnCaP alloy promotes cell activity and migration, providing favorable conditions for osteogenesis, thereby advancing the bone repair process.

To systematically explore the mechanism by which the Mg+FnCaP alloy promoted bone repair and osteogenic differentiation, we assessed its effect on the osteogenic differentiation of BMSCs using immunofluorescence analysis. The results showed that the expression levels of RUNX2 and OCN proteins in the Mg+FnCaP group were significantly higher than those in the other groups (Figure 6c, d), further validating the role of this material in promoting the osteogenic differentiation of BMSCs. Subsequently, we assessed the osteogenic differentiation of BMSCs in an osteoporotic microenvironment using alkaline phosphatase (ALP) staining and alizarin red S (ARS) staining. The ALP activity was measured at 7 and 14 days, and the results (Figure 6e, g, h) showed that the Mg+FnCaP group had significantly higher ALP activity than the other groups at both time points, indicating that this material has a significant effect on promoting the early osteogenic differentiation of BMSCs. ARS staining assessments at 14 and 21 d also showed (Figure 6f, i, j) that the calcium deposition area in the Mg+FnCaP group was significantly larger than that in the other groups, and calcium deposition continued to increase with prolonged incubation time, indicating that the material had a sustained promotional effect on BMSCs osteogenic differentiation. Additionally, RT‐qPCR analysis showed that the expression levels of osteogenesis‐related genes (RUNX2, OCN, Col‐I, and Osterix) in the Mg+FnCaP group were significantly higher than those in the other groups (Figure 6k–n). These results indicated that the Mg+FnCaP implant material significantly promoted the osteogenic differentiation of BMSCs. This material not only significantly enhanced the early osteogenic activity of BMSCs but also continuously promoted calcium deposition and bone matrix synthesis, demonstrating strong osteogenic induction capacity. Furthermore, the Mg+FnCaP implant material provides ideal support for BMSCs in the osteoporotic microenvironment by releasing Ca^2^⁺ and P ions, further enhancing the expression of osteogenesis‐related genes and proteins. More importantly, Mg+FnCaP effectively promoted BMSCs migration, helping overcome the adverse effects of the osteoporotic microenvironment. Furthermore, the direct osteogenic activity of BMSCs on different substrates was further investigated (Figure S17). Alkaline phosphatase (ALP) and alkaline staining (ARS) results indicated that the Mg+FnCaP group directly promoted the osteogenic differentiation of BMSCs, which was potentially attributable to the sustained release of magnesium ions and calcium phosphate within the Mg+FnCaP material. These findings suggest that the pronounced osteogenic effects induced by Mg+FnCaP materials result from the synergistic interaction between the direct osteogenic properties of magnesium ions and calcium phosphate, and their mediated optimization of the immunomodulatory microenvironment. Overall, the Mg+FnCaP implant material has great potential for application in bone tissue repair and regeneration by enhancing BMSCs migration and osteogenic capacity.

### 2.8 Osteogenesis Promotion by Pearl‐Like Coated Magnesium Alloy in the Osteoporotic Animal Model through Enhanced Immune‐Bone Growth Factor Cascades

**FIGURE 7:**
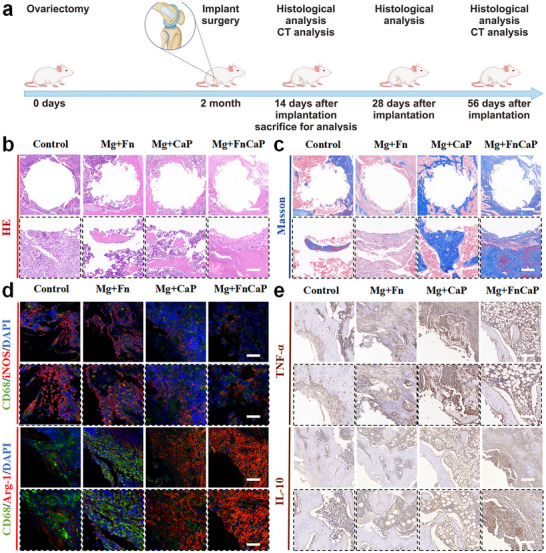
Mg+FnCaP regulated macrophage polarization in vivo. a) Scheme illustrating the modeling and animal experiment processing flow. b) H&E and c) Masson staining of the peri‐implant bone tissues at 2 weeks, scale bar = 200 µm (top), 25 µm (bottom) (n = 5). d) Immunofluorescence images of the peri‐implant tissue: red (M1 marker, iNOS; or M2 marker, Arg‐1), green (CD68, rat macrophage‐specific antigen marker), and blue (nuclei), scale bar = 50 µm (top) and 20 µm (bottom) (n = 5). e) Immunohistochemical staining of TNF‐α and IL‐10 in the peri‐implant tissue, scale bar = 50 µm (top) and 20 µm (bottom) (n = 5).

After completing the in vitro macrophage immunomodulation study, we evaluated the in vivo effects of Mg+FnCaP implants in an animal model of osteoporosis to explore their therapeutic potential further. We successfully established a rat model of osteoporosis using ovariectomy. The experimental procedure is illustrated in Figure 7a. We further evaluated the anti‐inflammatory effects to uncover the potential role of Mg+FnCaP implants in regulating inflammation associated with osteoporosis. To assess the anti‐inflammatory effects of Mg+FnCaP implants, we conducted a two‐week observation in an animal model and performed HE staining and Masson staining on the implanted tissue to evaluate its anti‐inflammatory effects comprehensively. The HE staining results (Figure 7b) showed that in the control group, there was significant infiltration of inflammatory cells in the implanted area, primarily monocytes, neutrophils, and a small number of lymphocytes, indicating that material implantation triggered a strong local inflammatory response. In contrast, the Mg+FnCaP group exhibited a significant reduction in inflammatory cell infiltration with a more intact tissue structure, demonstrating that this material possesses notable anti‐inflammatory effects and effectively suppresses local inflammatory reactions. Further analysis using Marson staining revealed (Figure 7c) that the Mg+FnCaP group exhibited a more compact collagen fiber structure, and the expansion of the inflammatory area was inhibited, with tissue recovery significantly superior to that of the control group. These results indicated that the Mg+FnCaP implant effectively alleviated pro‐inflammatory reactions and protected the tissue from excessive inflammatory damage. Additionally, the Mg+FnCaP group exhibited lower local swelling and tissue edema, indicating that the material had a sustained effect on alleviating local inflammation.

To validate these findings, we conducted immunofluorescence and immunohistochemical analyses to investigate the mechanism of action of this material on the inflammatory response. In immunofluorescence analysis, we observed a significant decrease in iNOS expression and a significant increase in Arg‐1 expression in the Mg+FnCaP group, indicating (Figure 7d; Figure S18) that this material regulates the immune response by inhibiting the production of pro‐inflammatory factors and promoting the expression of anti‐inflammatory factors, thereby providing a favorable immune environment for bone repair. To further evaluate the anti‐inflammatory effects of the Mg+FnCaP implant, we performed immunohistochemical analysis, focusing on changes in the expression of pro‐inflammatory factor TNF‐α and anti‐inflammatory factor IL‐10. Immunohistochemical results showed that TNF‐α expression levels were significantly elevated in the control group, indicating that material implantation triggered a significant pro‐inflammatory response. In contrast, TNF‐α expression was significantly reduced in the Mg+FnCaP group, indicating that this material effectively suppresses excessive pro‐inflammatory factor expression, thereby alleviating local inflammatory responses. Additionally, IL‐10 expression was significantly increased in the Mg+FnCaP group, indicating that this material not only enhanced anti‐inflammatory immune responses but also promoted self‐regulation of the immune system, thereby improving the immune microenvironment and providing strong support for tissue repair. These immunohistochemical results (Figure 7e; Figure S19) further confirm that Mg+FnCaP implants can effectively regulate local inflammatory responses, significantly reduce the expression levels of pro‐inflammatory factors such as TNF‐α, and increase the levels of anti‐inflammatory factors such as IL‐10, providing ideal immune support for bone repair. Overall, these findings strongly support the potential application of Mg+FnCaP in the treatment of osteoporosis, particularly due to its important role in regulating immune responses and promoting bone repair. The anti‐inflammatory effects of Mg+FnCaP implants provide a favorable immune microenvironment for subsequent bone repair and reconstruction, further demonstrating their significant application value in the treatment of osteoporosis.

**FIGURE 8:**
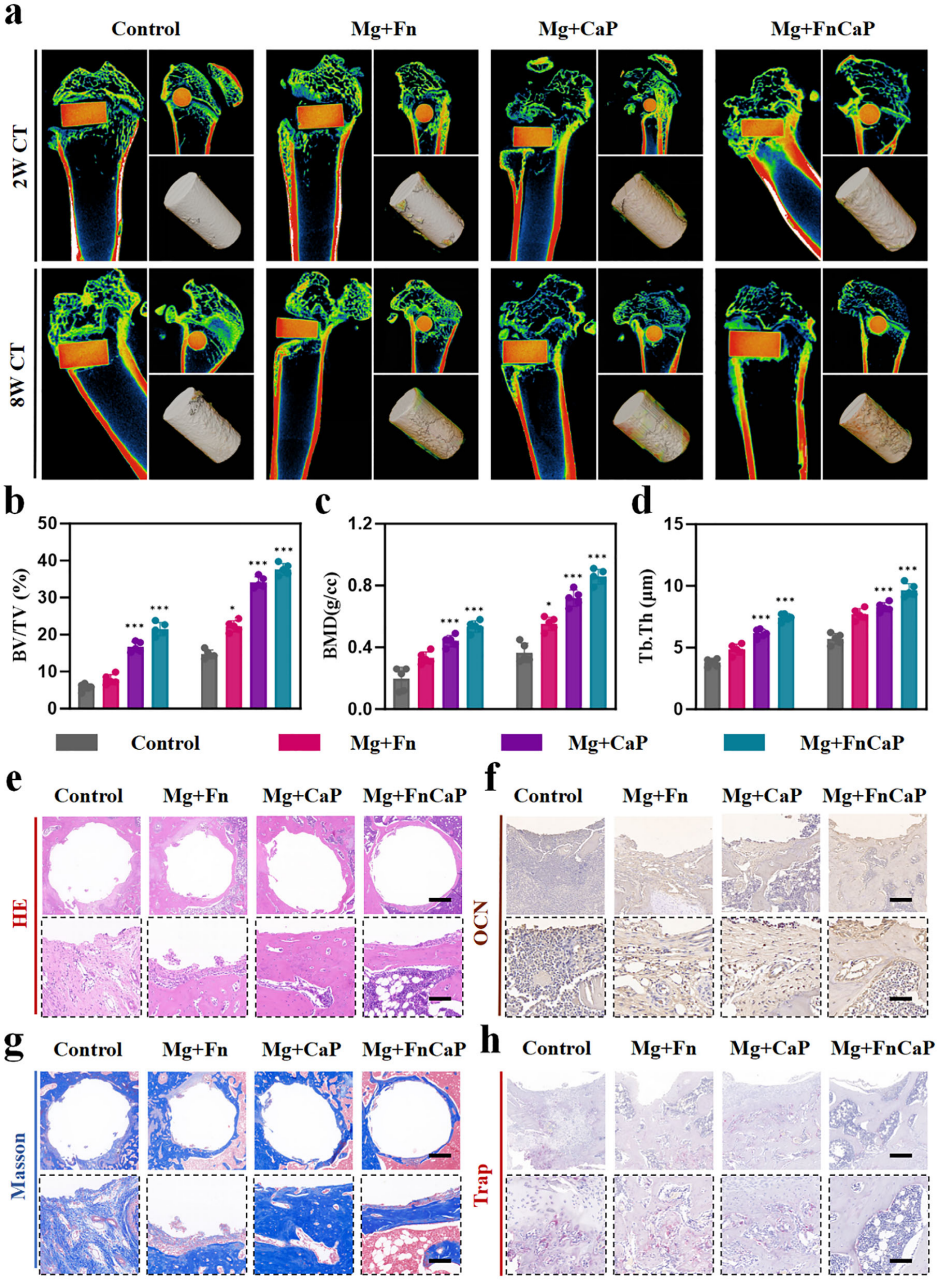
Mg+FnCaP promoted the osteointegration of the implant in vivo. a) Micro‐CT scanning and reconstructed 3D images of femurs containing different modified implantsat 2 weeks and 8 weeks, Spongy bone is predominantly yellowish‐green, cortical bone is mainly yellow, and the medullary cavity is black (n = 5). b–d) Quantitatively evaluating the peri‐implant bone generation according to BMD, BV/TV and Tb.Th (n = 5). e) H&E staining of the peri‐implant bone tissues, scale bar = 200 µm (top) and 25 µm (bottom) (n = 5). g) Masson staining of the peri‐implant bone tissues, scale bar = 200 µm (top) and 25 µm (bottom) (n = 5). f) Immunohistochemical staining of OCN in the peri‐implant tissue, scale bar = 200 µm (top) and 25 µm (bottom) (n = 5). h) TRAP staining in the peri‐implant tissue, scale bar = 200 (top) µm and 25 µm (bottom) (n = 5).Data shown represent the mean ± SD. Statistical analysis was performed using one‐way ANOVA with a Tukey's post hoc test. Compared with Control, *p < 0.05, ***p < 0.001, p > 0.05 were designated as not significant (ns).

To evaluate the osseointegration efficacy of Mg+FnCaP‐modified implants in diabetic rats, we conducted observations using micro‐CT scans and 3D reconstruction images and assessed bone formation around the implants at 2 and 8 weeks post‐implantation (Figure 8a). At 2 weeks, micro‐CT scans and 3D reconstructed images showed that bone formation around the plain Mg‐based implants was limited, with low bone mineral density, whereas Mg+FnCaP‐modified implants exhibited significantly more bone tissue, demonstrating markedly superior osseointegration compared with Mg‐based materials. After eight weeks of observation, the Mg+FnCaP group showed significantly more bone tissue formation, with bone mineral density and bone volume fraction around the implant being significantly higher than those in the control group, indicating that this material has a significant effect on promoting bone repair and osseointegration (Figure 8b–d). Further histological staining was performed using HE eosin and Masson staining. The results showed that in the control group, there was significant fibrous tissue infiltration around the implant with a thick fibrous layer, which exerted a significant inhibitory effect on the osteogenic process and impaired bone remodeling. In contrast, in the Mg+FnCaP group, the thickness of the fibrous layer was significantly reduced, bone tissue formation was markedly increased, tissue structure was more compact, and bone formation and osseointegration were more stable (Figure 8c, g). These histological results further confirmed the excellent biocompatibility and osseointegration capacity of the Mg+FnCaP implant. Additionally, we assessed dynamic changes in osteogenesis and bone resorption using OCN and Tartrate‐Resistant Acid Phosphatase (TRAP) staining. The OCN staining results (Figure 8f; Figure S20) showed that osteocalcin expression was significantly higher in the Mg+FnCaP group than in the control group, indicating that this material promotes osteoblast differentiation and bone matrix synthesis. The TRAP staining results (Figure 8h; Figure S20) indicated that the Mg+FnCaP group exhibited lower bone resorption activity, further supporting the idea that this material effectively balances bone resorption and formation while promoting bone formation, thereby facilitating healthier bone remodeling. These results indicate that Mg+FnCaP‐modified implants not only significantly enhance bone integration between the bone tissue and the implant but also provide ideal immune support for bone repair by promoting osteoblast activity, enhancing osteopontin expression, and inhibiting bone resorption. In addition, H&E staining of the major organs (heart, liver, spleen, lungs, and kidneys) showed no significant toxicity, suggesting that Mg+FnCaP is biologically safe in vivo (Figure S21). Overall, the Mg+FnCaP implants demonstrated promising potential for osteoporosis treatment, particularly for regulating immune responses and promoting bone repair processes.

## 3 Conclusion

Based on this study, the Mg+FnCaP coating system organically integrates Ca‐P mineral layers with fibronectin‐mimetic peptides (Fn‐mimetic peptides) through a biomimetic “brick–mortar” structure, constructing a functional interface on magnesium alloy surfaces that combines mechanical stability with biological activity. Ca‐P “bricks” significantly mitigate rapid corrosion of magnesium in body fluid environments, reduce local pH fluctuations and hydrogen gas release, and enhance coating structural integrity and fracture toughness. While the Fn‐mimetic peptide acts as a “mortar,” not only enhancing cell adhesion and osteoblast recruitment but also effectively establishing an immune‐vascular‐osteogenic microenvironment conducive to bone repair by modulating macrophage phenotypes and activating angiogenesis signaling pathways.

This pearl‐like‐coated magnesium alloy possesses anti‐inflammatory and antioxidant properties that help reduce inflammation and oxidative damage, thus ensuring stable support from the implant before complete bone healing. Its advantages lie in promoting bone regeneration, enhancing mechanical strength, and providing additional support in pathological conditions such as osteoporosis [50, 51]. It significantly enhances early osseointegration and suppresses inflammatory responses and oxidative stress, thereby enabling faster, more stable bone regeneration. In summary, the Mg+FnCaP coating not only addresses the issues of rapid corrosion and strong biological inertia in traditional magnesium alloy implants but also facilitates their transformation from passive mechanical scaffolds to active therapeutic platforms through the synergistic activation of multiple biological functions. This material system provides a robust technological foundation and broad clinical application prospects for the development of a new generation of intelligent internal fixation devices with degradability, elimination of secondary surgeries, immune regulation, and bone‐regeneration promotion capabilities.

## Conflicts of Interest

The authors declare no conflict of interest.

## Supporting information




**Supporting File**: advs73400‐sup‐0001‐SuppMat.docx.

## Data Availability

The data that support the findings of this study are available from the corresponding author upon reasonable request.
