# Supplementary material for: Pearl‐Like Bioinspired Coating Enables Regulation of Mg Degradation for Osteoporotic Bone Repair
Source: Adv Sci (Weinh). 2025 Dec 20;13(13):e21927. doi: 10.1002/advs.202521927 (PMC12955908; doi:10.1002/advs.202521927)
Supplement: Supplementary file 1 — Supporting File: advs73400‐sup‐0001‐SuppMat.docx. [file ADVS-13-e21927-s001.docx]

### Pearl-Like Bioinspired Coating Enables Regulation of Mg Degradation for Osteoporotic Bone Repair

*Siming Zhang,*^a1^ *Tao Zhang,*^a1^ *Yuan Chen,*^b1^ Nikolaos Kourkoumelis*,*^c^ *Mo Chen,*^a^ *Jiale Dong,*^a^ *Zhenyu Li,*^a^ *Yanling Zhou,*^b^ *Ning Li,*^a^ *Chen Zhu,^a^* Xifu Shang,*^a^** Jiaxiang Bai,*^a^*** *& Xianzuo Zhang,^a^**

^a^ Department of Orthopedics, Centre for Leading Medicine and Advanced Technologies of IHM, The First Affiliated Hospital of USTC, Division of Life Sciences and Medicine, University of Science and Technology of China, Hefei, Anhui, 230001, China.

^b^ Department of Orthopaedics, The Second Affiliated Hospital of Anhui University of Chinese Medicine, Hefei, Anhui, 230031, China.

^c^ Department of Medical Physics, University of Ioannina, Ioannina, Greece

* Correspondence and requests for materials should be addressed to

**Xianzuo Zhang**

Department of Orthopedics, Centre for Leading Medicine and Advanced Technologies of IHM, The First Affiliated Hospital of USTC, Division of Life Sciences and Medicine, University of Science and Technology of China, Hefei 230022, China.

**E-mail: [zhangxianzuo@ustc.edu.cn](mailto:zhangxianzuo@ustc.edu.cn)**

**Jiaxiang Bai**

Department of Orthopedics, Centre for Leading Medicine and Advanced Technologies of IHM, The First Affiliated Hospital of USTC, Division of Life Sciences and Medicine, University of Science and Technology of China, Hefei 230022, China.

**E-mail: [jxbai1995@ustc.edu.cn](mailto:jxbai1995@ustc.edu.cn)**

**Xifu Shang**

Department of Orthopedics, Centre for Leading Medicine and Advanced Technologies of IHM, The First Affiliated Hospital of USTC, Division of Life Sciences and Medicine, University of Science and Technology of China, Hefei 230022, China.

**E-mail: shangxifu@ustc.edu.cn**

**Chen Zhu**

Department of Orthopedics, Centre for Leading Medicine and Advanced Technologies of IHM, The First Affiliated Hospital of USTC, Division of Life Sciences and Medicine, University of Science and Technology of China, Hefei 230022, China.

**E-mail: zhuchena@ustc.edu.cn**

^1^ These authors contributed equally to this work.

**Supporting Information**

1. **Additional Experimental Methods**

**Synthesis of pearl-like Coating Materials**

During the hydrothermal treatment process, a solution of 14 mM calcium nitrate (Ca(NO_3_)_2_·4H_2_O), 8.4 mM sodium dihydrogen phosphate (NaH_2_PO_4_·2H_2_O), and 4 mM sodium bicarbonate (NaHCO_3_) is reacted, and the pH is adjusted to induce the precipitation of calcium phosphate crystals, forming a suspension. Next, a 0.01 mg/mL solution of 3-mercaptopropionic acid (MPA)-polyethylene glycol (PEG)-cyclic peptide is added and mixed with the calcium phosphate suspension. Freeze-thaw treatment is applied to ensure the uniform attachment of the coating to the magnesium alloy surface. This process is repeated to ensure the coating is firm, with a rough surface and high porosity. Subsequently, a layer of fibrinogen-like protein (synthesized by peptide synthesis methods) is first added to the magnesium alloy surface, followed by the repeated application of a calcium phosphate coating. This alternation of adding fibrinogen-like protein layers and calcium phosphate coatings is repeated several times to enhance the stability and effectiveness of the coating. Finally, a pressure of 10 kPa is applied to compress the coating, making it more compact and further improving its adhesion and performance.

**Synthesis of the Fn Peptide**

The Fn peptide was synthesized using a standard Fmoc solid-phase peptide synthesis (SPPS) strategy, with the sequence MPA-PEG-Glu{c|Arg-Gly-Asp-D-Phe-Lys(Ahx)|}-{c|Arg-Gly-Asp-D-Phe-Lys(Ahx)|}. Specifically, the synthesis commenced on Rink Amide MBHA resin, and two identical linear segments of "Arg-Gly-Asp-D-Phe-Lys(Ahx)" were sequentially assembled. A branching point was introduced using Fmoc-Glu-OAll, where the γ-carboxyl side chain was orthogonally protected with an allyl ester. Upon completion of the second peptide chain, the allyl ester protecting group on Glu was selectively removed using Pd(PPh₃)₄. This was followed by two sequential on-resin, head-to-tail cyclizations: the first between the exposed side chain γ-carboxyl of Glu and the α-amino group of the N-terminal Arg of the second peptide chain, and the second between the C-terminal carboxyl of the first peptide chain [from Lys(Ahx)] and the α-amino group of the N-terminal Arg of the second peptide chain. Both cyclization reactions were facilitated by the coupling reagents HATU/HOAt in the presence of DIPEA. After cyclization, a PEG spacer and mercaptopropionic acid (MPA) were conjugated sequentially at the N-terminus. The crude peptide was then cleaved from the resin simultaneously with global side-chain deprotection using a TFA/water/TIPS (95:2.5:2.5, v/v/v) cocktail. The resulting crude product was precipitated in cold diethyl ether, purified by preparative reverse-phase HPLC on a C18 column using a gradient of water/acetonitrile (each containing 0.1% TFA), and finally characterized by MALDI-TOF mass spectrometry, which confirmed a molecular mass consistent with the theoretical value, yielding the pure target peptide.

**SEM:** After ultrasonic cleaning with deionized water and absolute ethanol, samples—including magnesium alloy, magnesium alloy with calcium phosphate coating, and magnesium alloy with Fn-calcium phosphate coating—were dried and coated with a thin layer of gold. The surface morphology of each group was then examined by scanning electron microscopy (SEM). Analyses focused on coating uniformity, surface roughness, and pore structure. By comparing the microstructure of the samples before and after different surface modifications, the effectiveness of calcium phosphate and Fn-calcium phosphate coatings in enhancing the surface morphology and density of the magnesium alloy was systematically evaluated.

**XPS:** After cleaning and drying, the magnesium alloy, magnesium alloy with calcium phosphate coating, and magnesium alloy with Fn-calcium phosphate coating samples were analyzed using X-ray photoelectron spectroscopy (XPS). During the analysis, the binding energies and relative contents of Mg, Ca, P, O, C, N, and other relevant elements on the sample surfaces were recorded. High-resolution spectra of selected regions were further acquired and subjected to detailed peak fitting. By comparing the elemental composition and chemical states among the different groups, the structural characteristics and surface chemical modifications introduced by the calcium phosphate and Fn-calcium phosphate functional coatings were systematically elucidated.

**FTIR:** The three groups of materials—magnesium alloy, magnesium alloy with calcium phosphate coating, and magnesium alloy with Fn-calcium phosphate coating—were analyzed using Fourier transform infrared spectroscopy (FTIR) over a wavenumber range of 4000 to 400 cm⁻¹. The infrared absorption spectra of each sample were obtained to identify characteristic peaks corresponding to major functional groups, such as phosphate groups, carboxyl groups, and peptide bonds. By comparing the FTIR spectra of the different coatings, the introduction of surface functional groups and changes in the chemical structure of the materials were further confirmed.

**Magnesium Ion Concentration Detection:** Pure magnesium, magnesium alloy, magnesium alloy with calcium phosphate coating, and magnesium alloy with Fn-calcium phosphate coating were each immersed in phosphate-buffered saline (PBS) and incubated at 37°C under static conditions. At predetermined time points (e.g., 7, 14, 21, and 28 days), the immersion solutions were collected for analysis. The concentration of Mg²⁺ ions released into the solution was measured using inductively coupled plasma optical emission spectrometry (ICP-OES). By plotting ion release profiles, the effect of different surface modifications on the Mg²⁺ release rate and sustained-release properties of the magnesium alloy was systematically evaluated. These results provided experimental evidence for the biodegradation behavior and controlled release mechanisms of the coatings.

**Water contact angle:** Samples of magnesium alloy, magnesium alloy with calcium phosphate coating, and magnesium alloy with Fn-calcium phosphate coating were cut into small pieces (1 cm × 1 cm) and ultrasonically cleaned in deionized water and absolute ethanol for 10 minutes each, then dried for further use. Each sample was placed flat on the stage of a contact angle measuring instrument (e.g., KRÜSS DSA30) at room temperature. Using a microsyringe, a 2 μL droplet of deionized water was carefully deposited onto the surface of each sample. After the droplet stabilized, the instrument automatically captured the shape of the droplet on the sample surface, and the contact angle was measured and recorded using the accompanying software. For each group, at least five different locations on the sample were tested, and the average contact angle was calculated for comparative analysis. By comparing the water contact angles of the three groups, the effect of different surface modifications on the wettability and hydrophilicity of the material surfaces was evaluated.

**Tensile strength, compressive strength:** To evaluate the mechanical properties of the magnesium alloy, magnesium alloy with calcium phosphate coating, and magnesium alloy with Fn-calcium phosphate coating, samples from each group were machined into standard tensile specimens and subjected to tensile testing using a universal testing machine at room temperature. Specimens were stretched at a specified loading rate until failure, and stress-strain curves were automatically recorded. The maximum stress prior to fracture was defined as the ultimate tensile strength of each sample. For compressive strength testing, standard cylindrical or cubic specimens were prepared and tested using a universal testing machine. Samples were loaded at a constant rate between the compression plates until obvious structural failure occurred, and the maximum load at failure was recorded. Compressive strength was calculated based on the recorded maximum force and the cross-sectional area of the specimen.

**Degradation rate:** The degradation behavior of the samples was evaluated through an immersion test. The initial mass of each sample was accurately measured, after which the samples were immersed in simulated body fluid (SBF) at 37°C. After 14 days, the samples were removed, rinsed with deionized water and absolute ethanol, and dried to a constant weight before the final mass was recorded. The degradation rate was calculated based on the change in mass, the sample surface area, and the immersion time. Parallel specimens were included in each group, and the average value was used to ensure accurate degradation data.

**Electrochemical corrosion:** To comprehensively evaluate the electrochemical corrosion behavior of magnesium alloy, magnesium alloy with calcium phosphate coating, and magnesium alloy with Fn-calcium phosphate coating, multiple electrochemical testing methods were employed.Firstly, potentiodynamic polarization tests were conducted using a three-electrode system to obtain polarization curves for each sample. Corrosion potential and corrosion current density were determined, allowing for a comparative assessment of the corrosion resistance among the different materials. Secondly, electrochemical impedance spectroscopy (EIS) was performed at open circuit potential to record the impedance spectra over a wide frequency range. By fitting the data, charge transfer resistance and interfacial characteristics were analyzed to evaluate the protective performance of the coatings and the corrosion processes involved. Additionally, open circuit potential (OCP) measurements were carried out to continuously monitor the spontaneous potential changes of the samples in electrolyte solution, providing insights into their surface stability and corrosion tendency. Finally, cyclic voltammetry (CV) was performed by repeatedly scanning the samples within a defined potential window. Analysis of the redox peaks and changes in the curves offered further understanding of the electrochemical reaction mechanisms occurring on the material surfaces. Through the combination of these four electrochemical techniques, the corrosion resistance and underlying mechanisms of the different materials and their coatings in simulated body fluid environments were systematically compared and evaluated.

**Cell culture:** Mouse macrophage RAW 264.7 and HUVECs were cultured in Dulbecco's Modified Eagle's Medium (DMEM), and preosteoblast bone marrow mesenchymal stem cells (BMSCs) were cultured in α-modified Eagle's medium (α-MEM), both supplemented with 10% foetal bovine serum (FBS), 1% penicillin and streptomycin, and cultured in a 90% humidity, 5% CO_2_, and 37°C incubator.

**Biocompatibility:** To evaluate the in vitro biosafety of magnesium alloy, magnesium alloy with calcium phosphate coating, and magnesium alloy with Fn-calcium phosphate coating, extract solutions from each sample were prepared and used for the culture of RAW264.7 cells, human umbilical vein endothelial cells (HUVECs), and BMSCs. Cell viability was assessed using the CCK-8 assay kit (Dojindo, Japan) according to the manufacturer’s instructions. For live/dead cell staining, cells were seeded onto glass coverslips in 24-well plates at a density of 20,000–30,000 cells per well. After incubation with the respective material extracts for the designated time, cell viability was further evaluated using a commercial Live/Dead® cell viability/cytotoxicity assay kit (Invitrogen, USA), following the supplier’s protocol. The effects of different sample extracts on cell proliferation and viability, as revealed by CCK-8 and live/dead staining results, were used to comprehensively assess the biocompatibility of the materials.

**Real-Time Quantitative PCR Assay：**Total RNA was extracted from cells in each experimental group using TRIzol reagent (Beyotime, China) according to the manufacturer’s instructions. The concentration and purity of the isolated RNA were determined spectrophotometrically. Subsequently, complementary DNA (cDNA) was synthesized from 1 μg of total RNA using a reverse transcription kit, following the provided protocol. Quantitative real-time PCR (qPCR) was then performed using the CFX96™ Real-Time PCR System (Bio-Rad, USA). Specific primers for the target genes are detailed in **Table S1** of the Supporting Information. The relative mRNA expression levels were calculated using the 2^−ΔΔCt method, with GAPDH serving as the internal control. All experiments were conducted in triplicate to ensure data reliability.

**Cellular Immunofluorescence Staining：**Cells were seeded onto sterile glass coverslips and subjected to different treatment conditions. After the interventions, cells were fixed in 4% paraformaldehyde for 20 minutes at room temperature and permeabilized with 0.2% Triton X-100 (Beyotime, China) for 10 minutes. The samples were then incubated overnight (at least 16 hours) at 4°C with the appropriate primary antibody. Following primary antibody incubation, cells were washed and incubated with the corresponding fluorescently labeled secondary antibody for 1 hour at room temperature in the dark. To visualize the cytoskeleton and nuclei, cells were further stained with ghost pen cyclic peptide (Yeasen, China) and DAPI (Beyotime, China), respectively. Fluorescent images were acquired using a fluorescence microscope (Zeiss, Germany). Quantitative analysis of fluorescence intensity was performed using ImageJ software (NIH, Bethesda, USA) to ensure objective assessment.

**Wound Healing Assay for Angiogenesis Evaluation：**To evaluate the angiogenic potential during wound healing, HUVECs (4 × 10⁵ cells/well) were seeded into 6-well plates and incubated at 37°C with 5% CO₂. Once the cells reached approximately 90% confluence, a scratch was made through the cell monolayer using a 200 μL pipette tip. The wells were then rinsed three times with sterile PBS to remove detached cells and debris. Subsequently, the medium was replaced with 0.5% serum and supplemented with conditioned medium derived from each hydrogel group. After 24 hours of incubation, images were captured using an inverted microscope. The area of cell migration was quantified using Image J software.

**Tube Formation Assay：**To further assess the angiogenic capacity of the hydrogels, 100 μL of Matrigel was added to each well of a 24-well plate and incubated at 37°C for 30 minutes to allow gelation. HUVECs (4 × 10⁴ cells/well) were then seeded onto the Matrigel in the presence of conditioned medium from different hydrogel groups. After incubation at 37°C with 5% CO₂ for various time points, the formation of capillary-like structures was imaged with a light microscope and analyzed using ImageJ.

**Transwell Migration Assay：**Cell migration was further evaluated using Transwell plates (Corning). Hydrogel extracts from each group were added to the lower chamber, while 200 μL of HUVECs suspension (3 × 10⁵ cells/mL) was added to the upper chamber. Following incubation at 37°C in 5% CO₂ for 16 hours, non-migrated cells on the upper surface of the membrane were removed with a cotton swab. The migrated cells were fixed with 4% paraformaldehyde for 20 minutes and stained with 0.1% crystal violet solution (Solarbio, Beijing, China). Images were acquired using a microscope, and the number of migrated cells was quantified with ImageJ.

**ALP staining and alizarin red S staining:** Alkaline phosphatase (ALP) staining was performed to evaluate the osteogenic differentiation capacity of BMSCs. Briefly, BMSCs were seeded in 24-well plates at a density of 40,000 cells per well and cultured in osteogenic induction medium for 7days and 14 days. Cells were then fixed with 4% paraformaldehyde and incubated with BCIP/NBT working solution (Beyotime Biotechnology, Shanghai, China) protected from light. ALP activity was further quantified using an alkaline phosphatase assay kit (Beyotime Biotechnology, Shanghai, China) according to the manufacturer's protocol, and absorbance was measured at 520 nm. For alizarin red S (ARS) staining, BMSCs were seeded in 24-well plates at a density of 20,000 cells per well and treated under different experimental conditions. After 14 days and 21 days of culture in osteogenic induction medium, cells were fixed with 4% paraformaldehyde and stained with 0.1% ARS solution (pH 4.2, Beyotime, China) in the dark. The formation of mineralized calcium nodules was observed under a light microscope (Zeiss, Germany). For quantification, the stained ARS was dissolved in 5% perchloric acid, and the optical density was measured at 490 nm.

**BMSCs extraction and** **exosome separation**

Primary BMSCs were isolated from 6-week-old Sprague-Dawley (SD) rats. After anesthesia, the rats were sacrificed, and femurs and tibias were collected. Muscles and soft tissues were carefully removed under sterile conditions. The bones were immersed in ethanol for 3 min for surface sterilization, rinsed repeatedly with sterile PBS, and the bone marrow cavity was flushed with culture medium into a petri dish. Cells were cultured in a 5% CO₂ incubator at 37 °C, and by replacing the medium regularly, pure BMSCs were gradually obtained.

**Animal model**

All experiments were approved by the Ethics Committee of the First Affiliated Hospital of the University of Science and Technology of China and conducted in accordance with the Guidelines for the Care and Use of Laboratory Animals **(2024-N(A)-0372)**. SD rats used in animal experiments were purchased from the Laboratory Animal Center of the University of Science and Technology of China (Hefei, China). To establish a castration-induced osteoporosis model, 5-week-old female SD rats weighing approximately 100 grams were selected. After one week of acclimation feeding, bilateral ovariectomy (OVX) was performed under anesthesia to induce the osteoporosis model. Postoperative care included routine housing and antibiotic administration for infection prevention.

**In vivo bone regeneration experiments:** To investigate the effects of various implants on osteogenesis in osteoporotic rats, an osteoporosis rat bone defect model was established. Forty female SD rats (200–220g, 8 weeks old) were randomly divided into four groups (n = 10 per group): magnesium alloy (Mg), Mg+Fn, Mg+CaP, and Mg+FnCaP. After the successful induction of osteoporosis, all rats were anesthetized with 3% sodium pentobarbital. The surgical area around the knee joint was shaved, disinfected, and a longitudinal incision was made. A standardized bone defect (3 mm in diameter and 5 mm in depth) was created at the lateral femoral condyle using a minimally invasive technique. The defect site was then thoroughly irrigated with sterile saline to remove any blood and debris. Subsequently, the designated implant for each group was carefully inserted into the bone defect. If required, the implantation site was exposed to ultraviolet light for 10–20 seconds. After implantation, the incisions were sutured, and penicillin was administered to prevent postoperative infection. At the designated endpoints, rats were euthanized, and femoral specimens were harvested for further analysis.

**Micro-CT analysis:** At 2 and 8 weeks after surgery, rats were euthanized and femurs were harvested. The femurs were fixed in 10% formaldehyde and analyzed using a SkyScan 1176 micro-CT scanner (Bruker, Belgium) with a resolution of 18 μm, at 65 kV and 500 μA. The region of interest (ROI) was defined as a cylindrical area with a diameter of 4 mm and a length of 8 mm, encompassing the bone defect site and the implant. For peri-implant bone analysis, a zone of interest (ZOI) was set as a 1 mm ring surrounding the implant surface. Three-dimensional reconstruction was used to evaluate bone regeneration around the implants. Quantitative measurements—including bone volume fraction (BV/TV), bone mineral density (BMD), bone density, trabecular number (Tb.N), and trabecular thickness (Tb.Th)—were calculated within the ZOI to assess the quality and extent of peri-implant bone formation.

**Histological and immunohistochemical analyses:** At the predetermined time points, femoral bones were isolated from osteoporotic rats, fixed with paraformaldehyde, subsequently decalcified and embedded in paraffin, and sectioned for histological analysis. Osteogenesis in the defect area was assessed by Hematoxylin and eosin (H&E) and Masson's trichrome staining, which provided evaluations of new bone formation, matrix composition, and the cellular dynamics of osteoblasts and osteoclasts.For immunofluorescence analysis, tissue sections were stained with antibodies against iNOS and Arg-1 to identify and assess macrophage polarization status in the wound microenvironment. Immunohistochemical staining was performed using antibodies against TNF-α, IL-10, and osteocalcin (OCN) to assess the expression levels of inflammatory and osteogenic markers in the tissue sections. Additionally, tartrate-resistant acid phosphatase (TRAP) staining was conducted to detect and quantify osteoclast activity. Briefly, after deparaffinization and rehydration, tissue sections were incubated with TRAP staining solution according to the manufacturer's instructions. TRAP-positive multinucleated cells were observed and counted under a light microscope to evaluate osteoclastogenesis and bone resorption activity within the wound tissue.

**Statistical Analysis:** Data are presented as the mean ± standard deviation (SD) from at least three independent experiments. For comparisons between two groups, an unpaired Student’s t-test was performed; for comparisons involving three or more groups, one-way analysis of variance (ANOVA) followed by Tukey’s post hoc test was applied. All statistical analyses were conducted using R software (version 4.3.1), and graphs were generated with the ggplot2 package within the R environment. A p-value of less than 0.05 was considered statistically significant, with specific levels denoted as *p < 0.05, **p < 0.01, and ***p < 0.001; results with p > 0.05 were designated as not significant (ns).

1. **Figures**

**
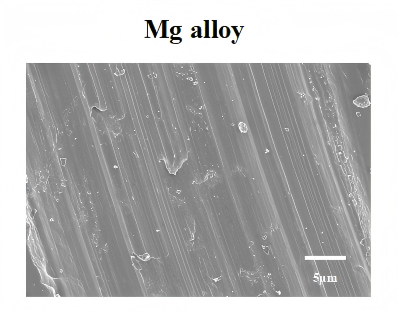
**

**Figure S1.** Representative scanning electron microscopy (SEM) images of the Mg alloy, scale bar = 5 μm.


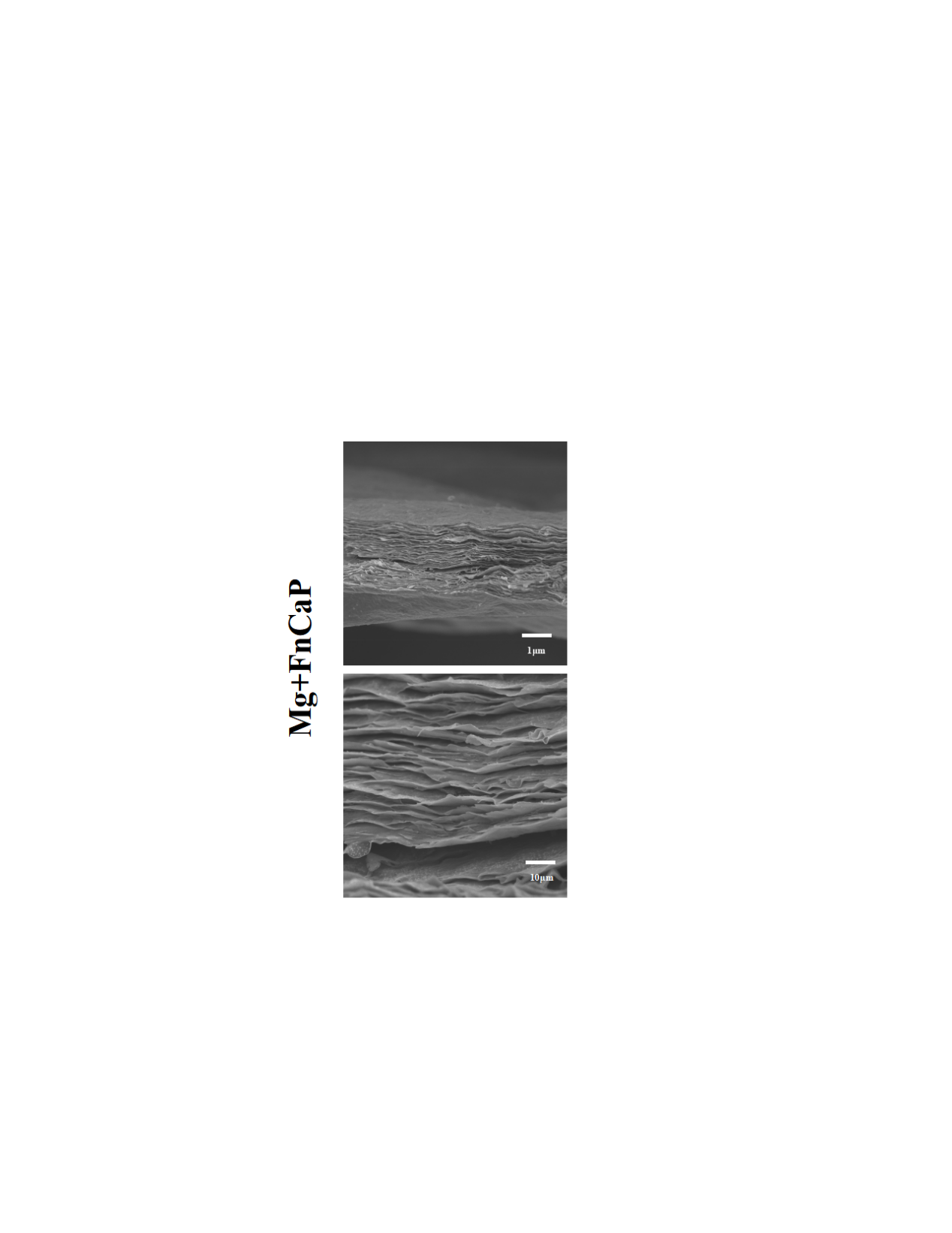


**Figure S2.** Representative cross-sectional SEM image of Mg+FnCaP.Scale bar = 1 µm (top), 10 µm (bottom).


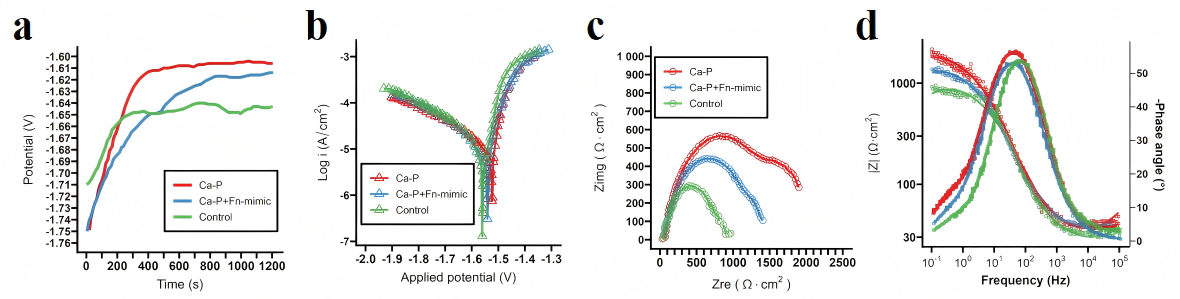


**Figure S3.** Electrochemical test results (immersed in Hank’s solution): (a) Potential–time curve (n = 3), (b) Potentiodynamic polarization curve (n = 3), (c) Nyquist plot (n = 3), (d) Bode plot. Control: magnesium alloy (n = 3).


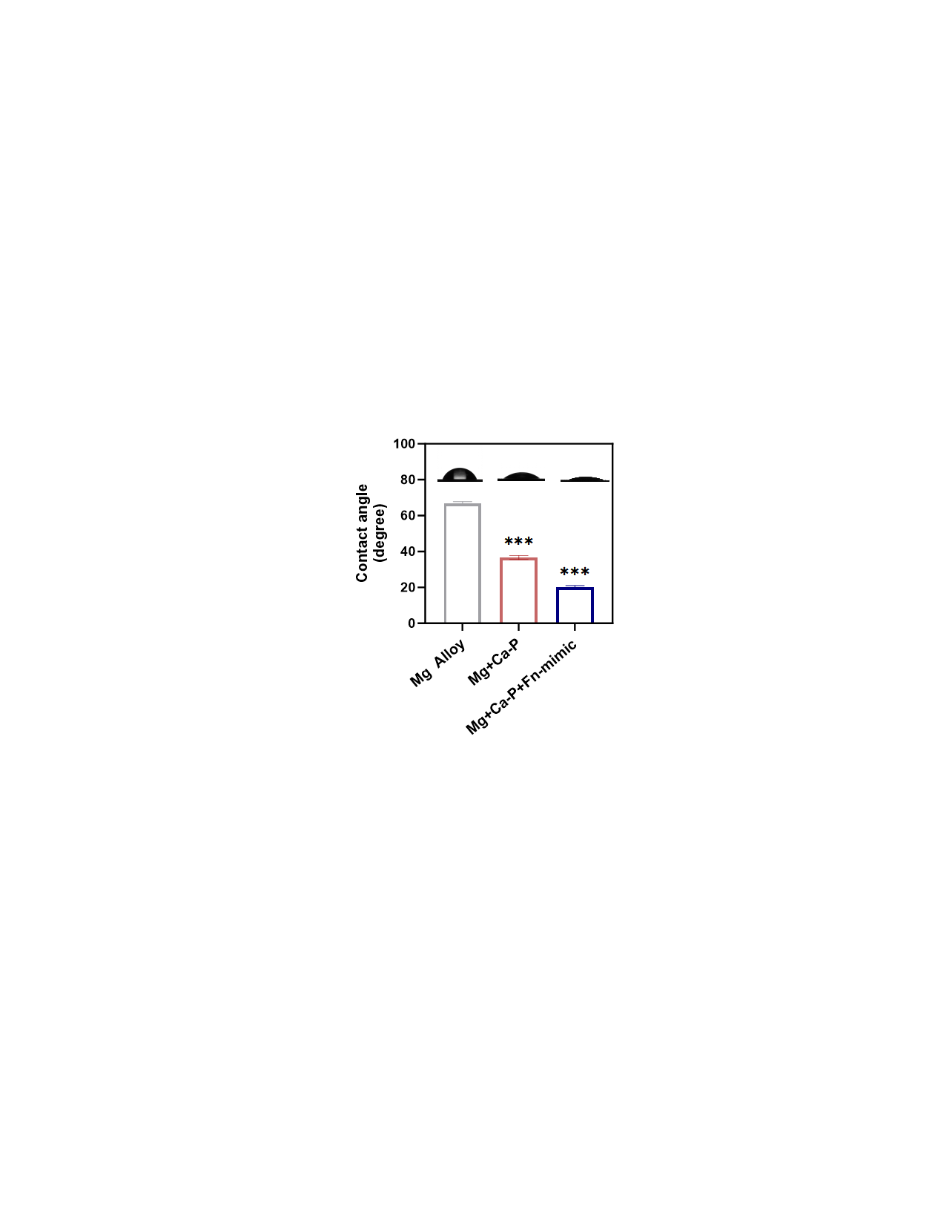


**Figure S4.** Contact angles of different substrates (n = 3). Data shown represent the mean ± SD. Statistical analysis was performed using one-way ANOVA test with a Tukey’s post hoc test. Compared with Control, ***p < 0.001.

**
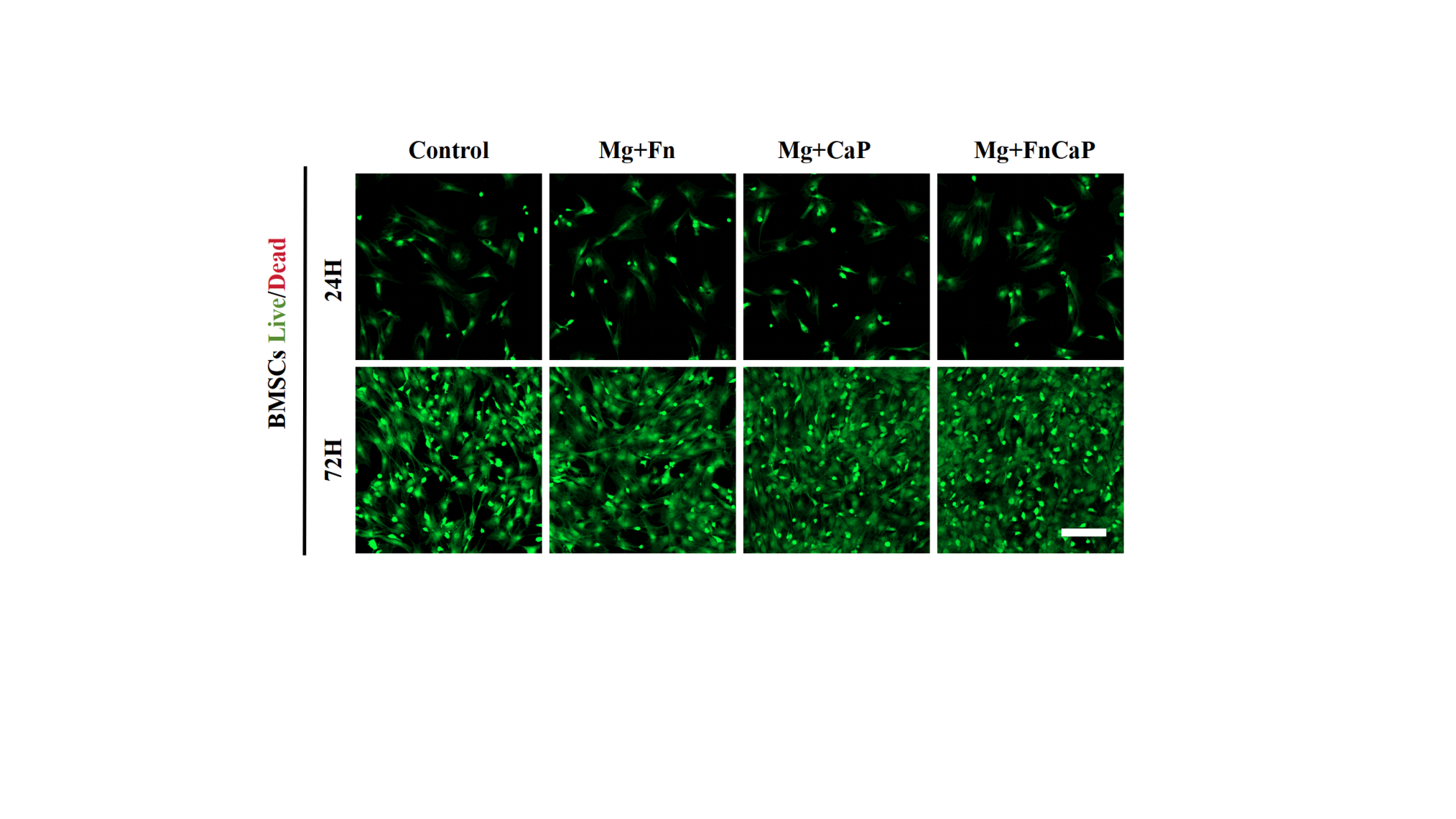
**

**Figure S5.** Live/dead staining results of BMSCs cells after different sample interventions, scale bar = 50 µm (n = 3).


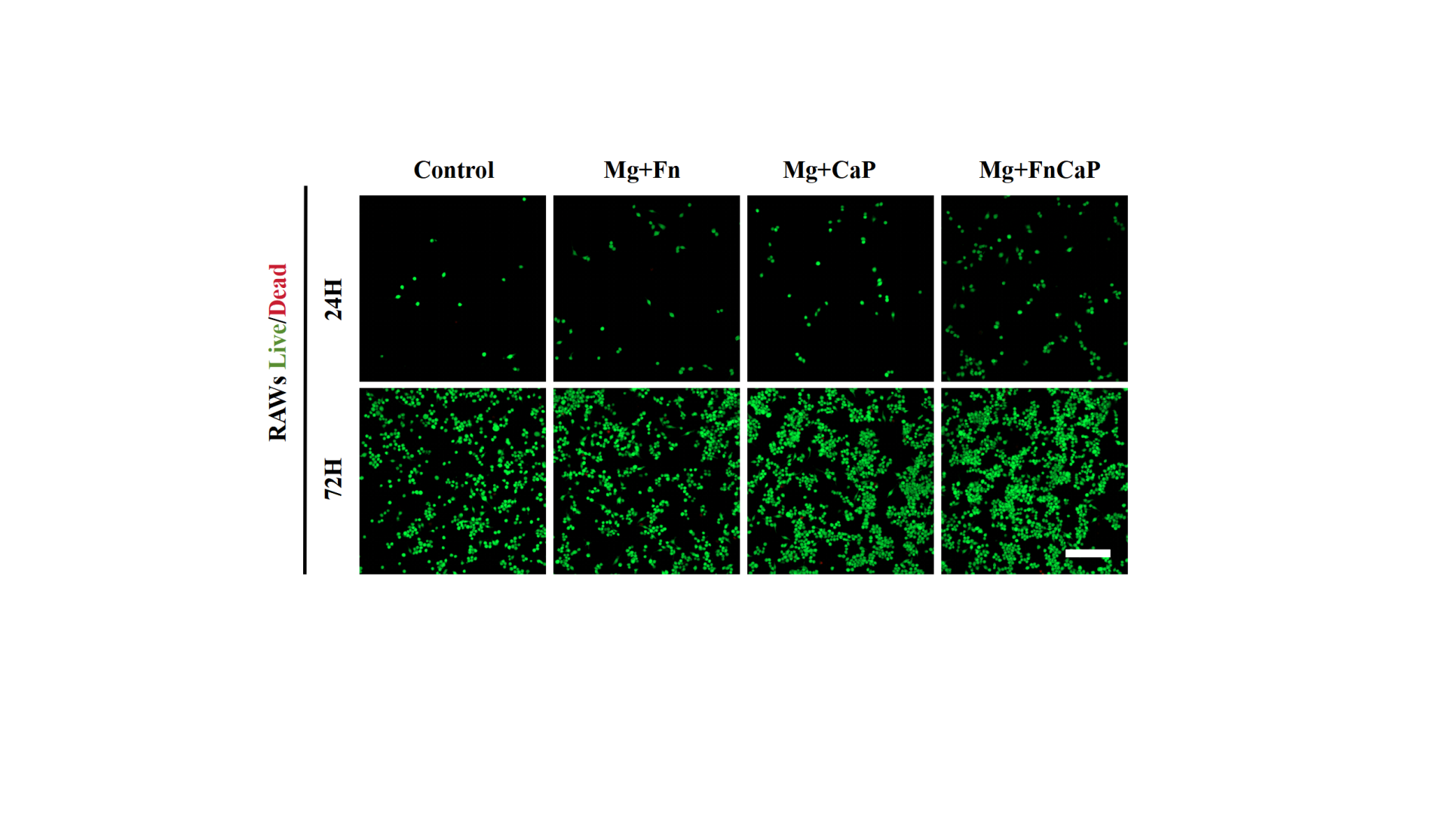


**Figure S6.** Live/dead staining results of RAW264.7 cells after different sample interventions, scale bar = 50 µm (n = 3).


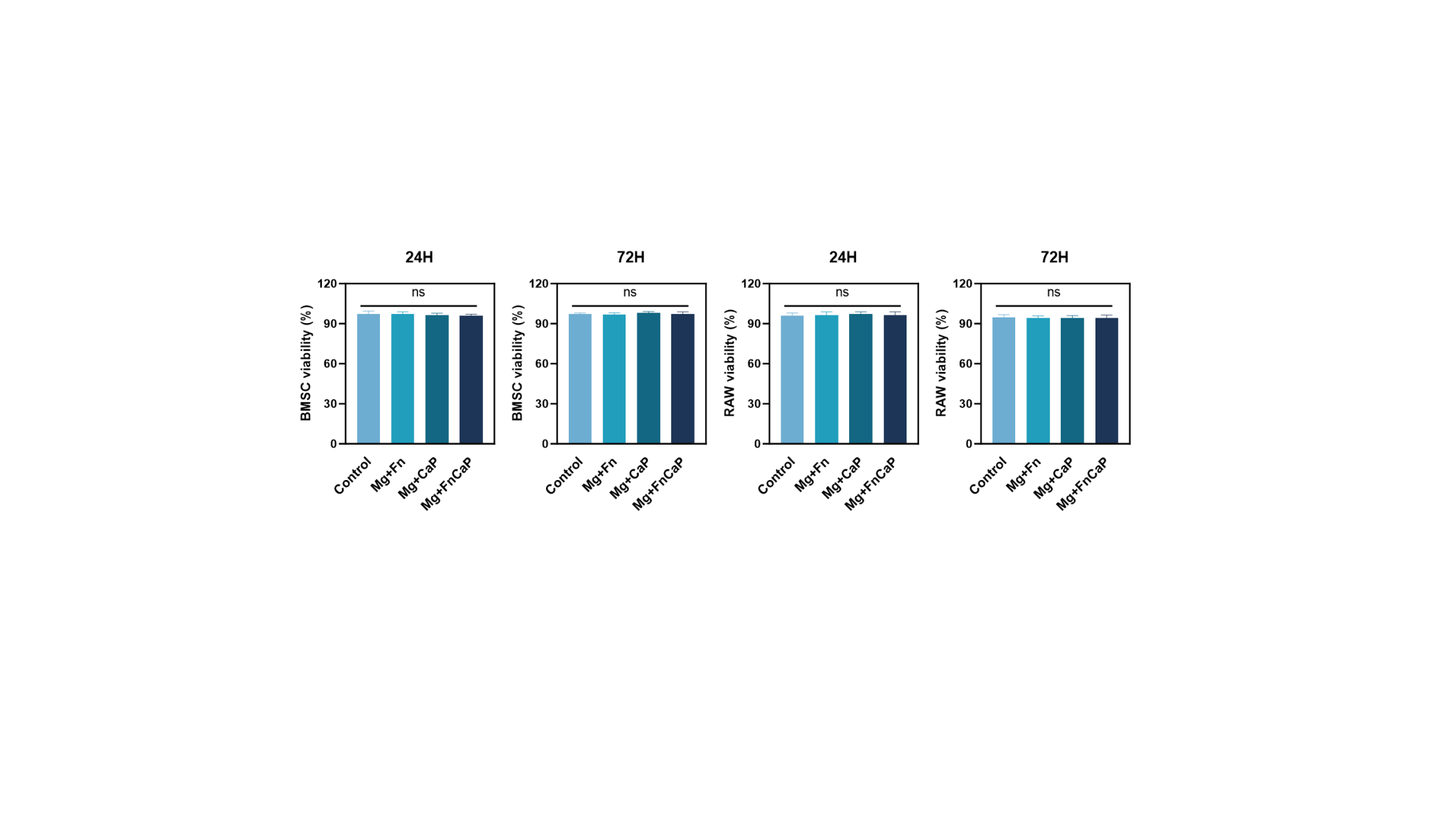


**Figure S7.** Corresponding quantitative data for Figure S6, 7 (n = 3). Data shown represent the mean ± SD. Statistical analysis was performed using one-way ANOVA test with a Tukey’s post hoc test. Compared with Control, p > 0.05 were designated as not significant (ns).


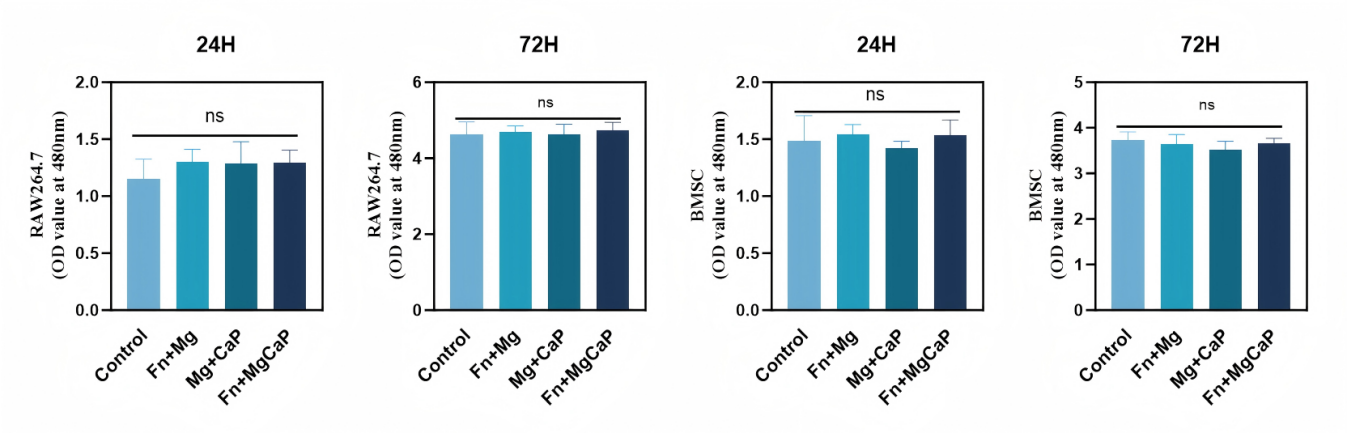


**Figure S8.** CCK-8 assay of BMSCs and RAW264.7 cells cultured on different surfaces for 1day and 3 days (n = 3). Data shown represent the mean ± SD. Statistical analysis was performed using one-way ANOVA test with a Tukey’s post hoc test. Compared with Control, p > 0.05 were designated as not significant (ns).


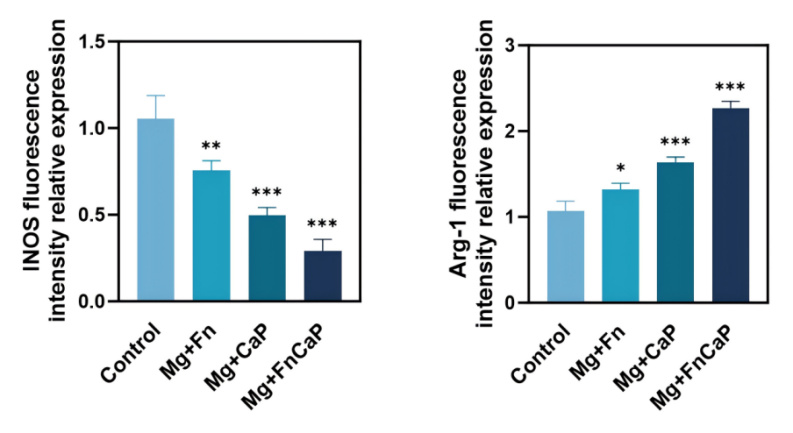


**Figure S9.** Corresponding quantitative data for Figure 2b (n = 3). Data shown represent the mean ± SD. Statistical analysis was performed using one-way ANOVA test with a Tukey’s post hoc test. Compared with Control, *p < 0.05, **p < 0.01, ***p < 0.001.


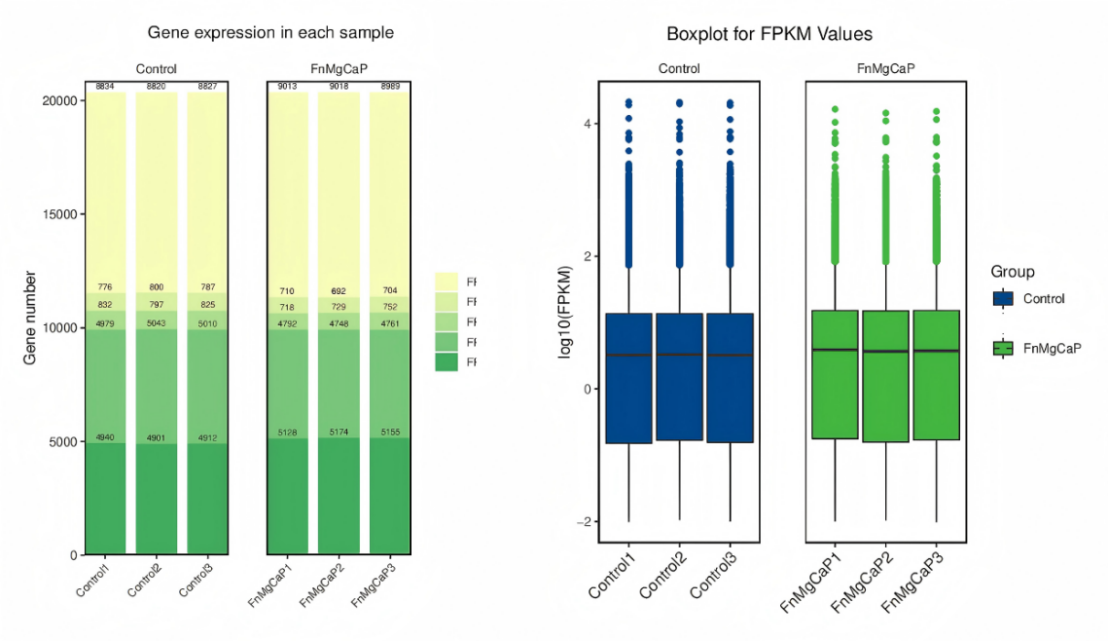


**Figure S10.** Gene expression and reads per kilobase million value (RPKM) in each sample of macrophages in Control and Mg+FnCaP treated groups according to RNA-seq results (n = 3).


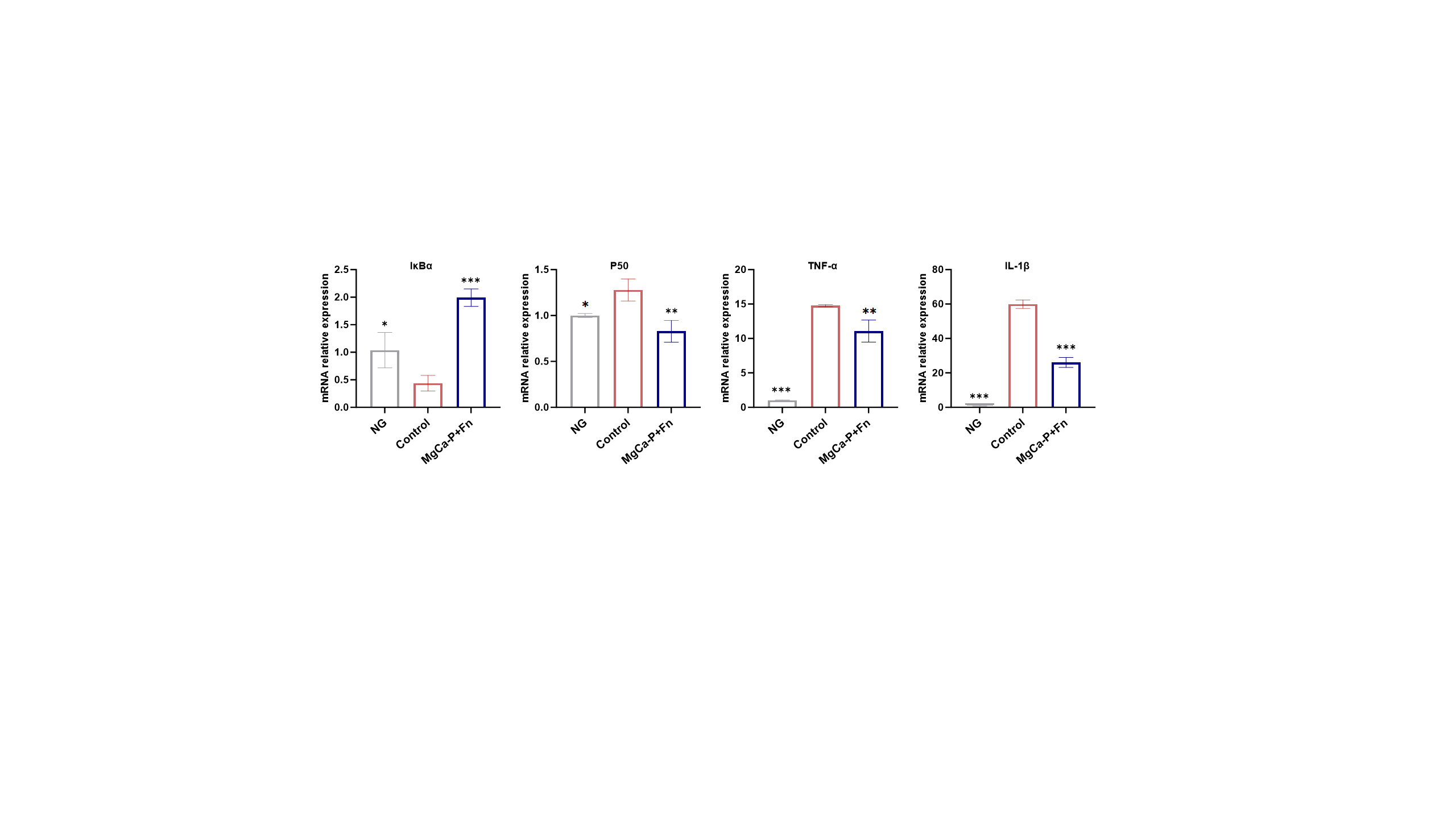


**Figure S11.** RT-qPCR results of IKBα, P50, TNF-α, and IL-1β expression in RAW264.7 cells (n = 3). Data shown represent the mean ± SD. Statistical analysis was performed using one-way ANOVA test with a Tukey’s post hoc test. Compared with Control, *p < 0.05, **p < 0.01, ***p < 0.001.


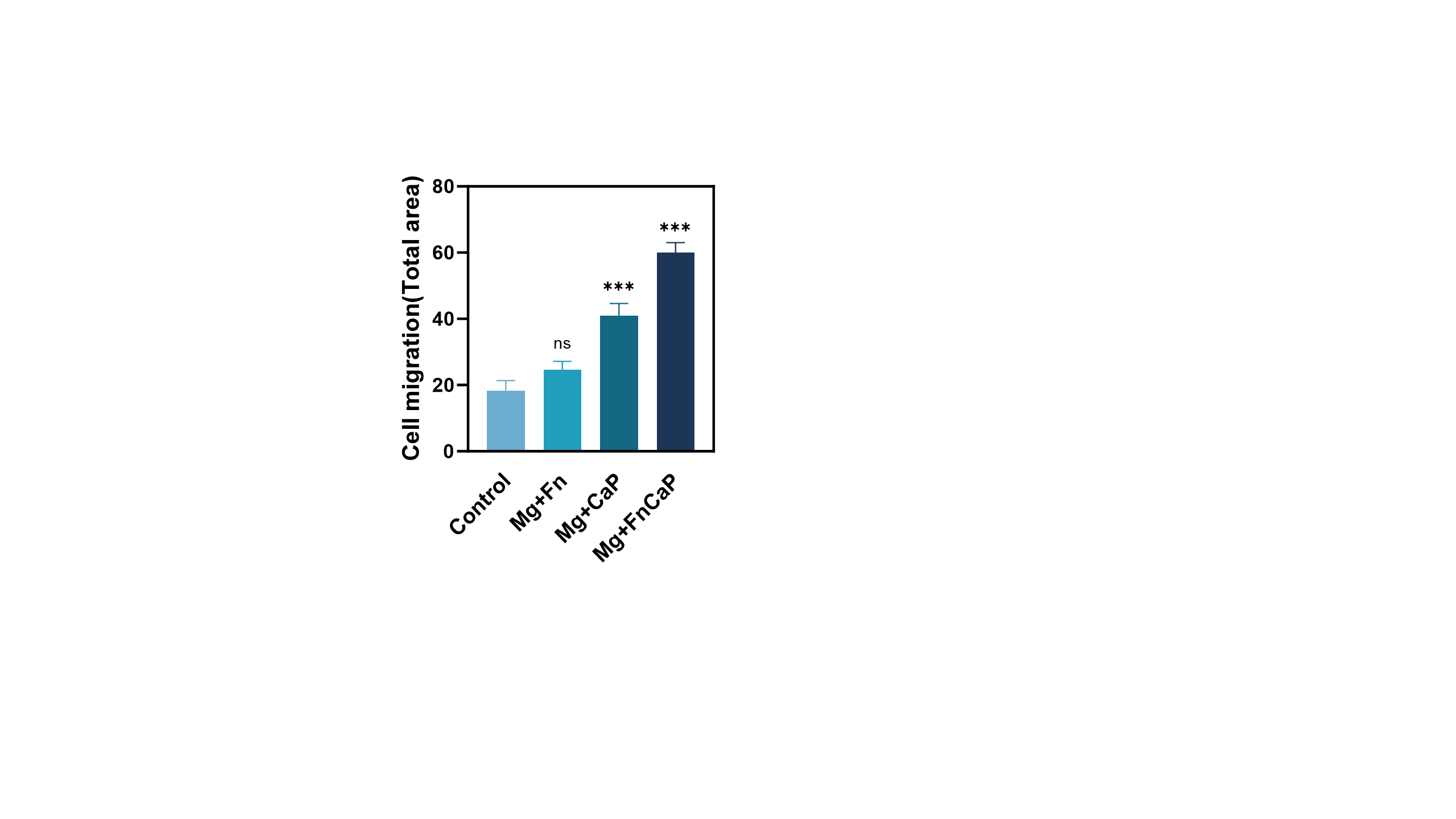


**Figure S12.** Corresponding quantitative data for Figure 5a (n = 3). Data shown represent the mean ± SD. Statistical analysis was performed using one-way ANOVA test with a Tukey’s post hoc test. Compared with Control, *p < 0.05, **p < 0.01, ***p < 0.001, p > 0.05 were designated as not significant (ns).


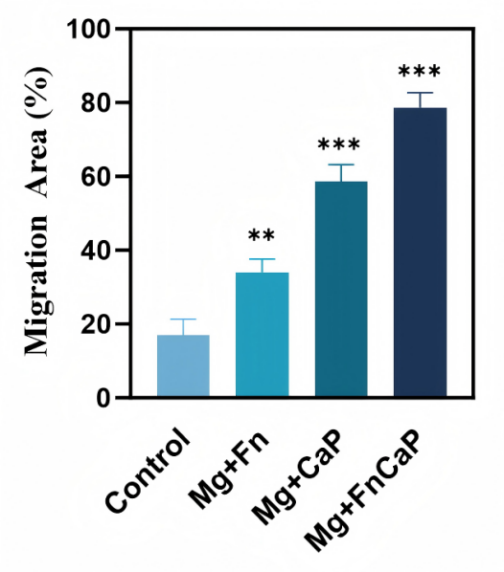


**Figure S13.** Corresponding quantitative data for Figure 5b (n = 3). Data shown represent the mean ± SD. Statistical analysis was performed using one-way ANOVA test with a Tukey’s post hoc test. Compared with Control, *p < 0.05, **p < 0.01, ***p < 0.001.


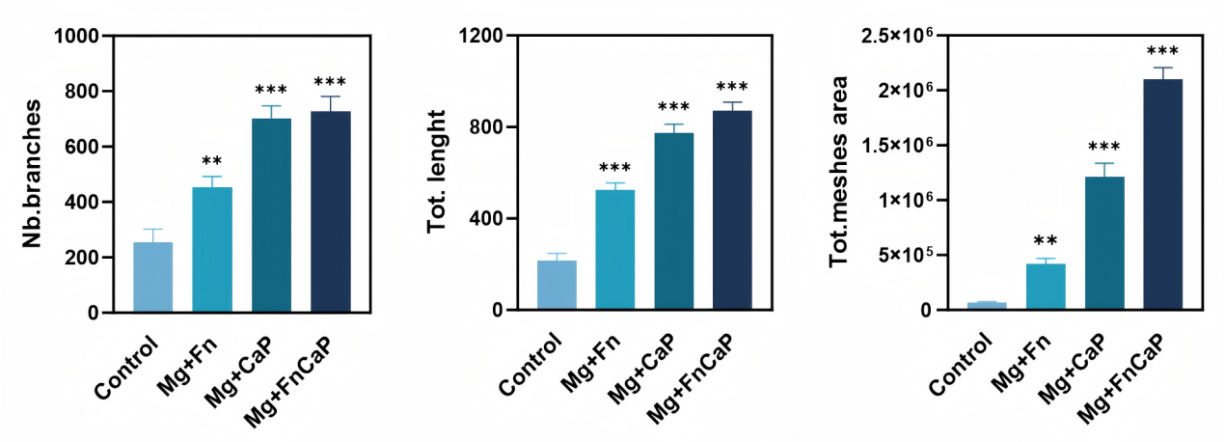


**Figure S14.** Corresponding quantitative data for Figure 5c (n = 3). Data shown represent the mean ± SD. Statistical analysis was performed using one-way ANOVA test with a Tukey’s post hoc test. Compared with Control, *p < 0.05, **p < 0.01, ***p < 0.001.


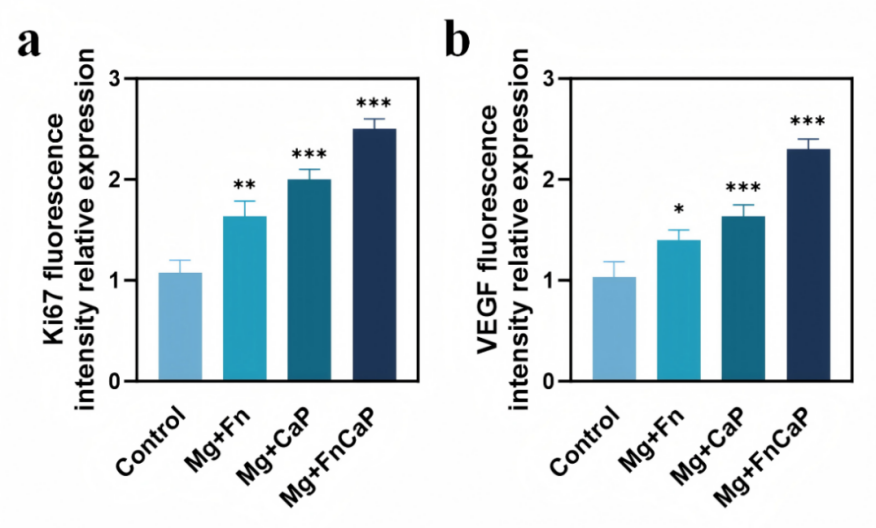


**Figure S15.** Corresponding quantitative data for Figure 5d, e (n = 3). Data shown represent the mean ± SD. Statistical analysis was performed using one-way ANOVA test with a Tukey’s post hoc test. Compared with Control, *p < 0.05, **p < 0.01, ***p < 0.001.


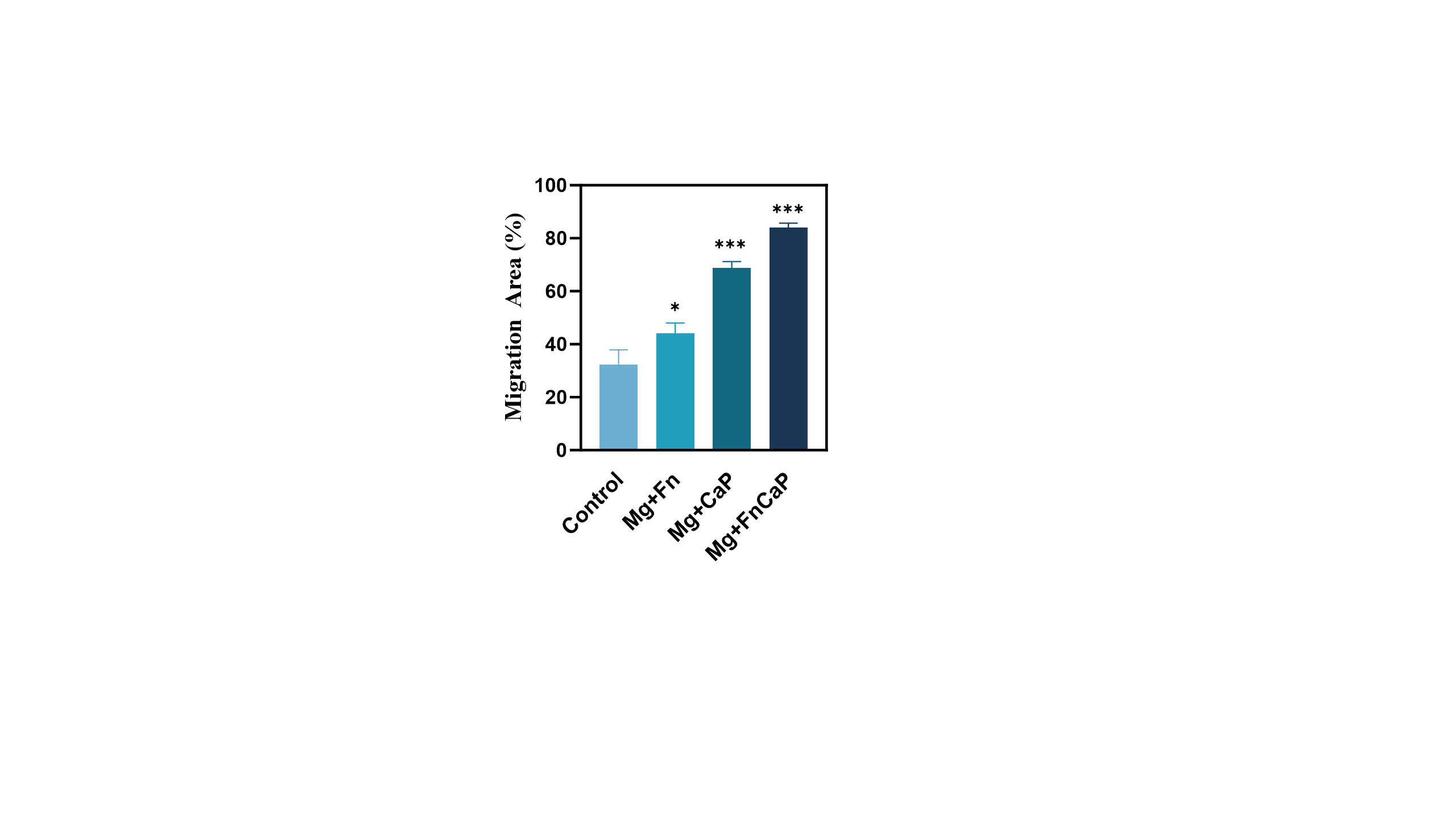


**Figure S16.** Corresponding quantitative data for Figure 6b (n = 3). Data shown represent the mean ± SD. Statistical analysis was performed using one-way ANOVA test with a Tukey’s post hoc test. Compared with Control, *p < 0.05, **p < 0.01, ***p < 0.001.


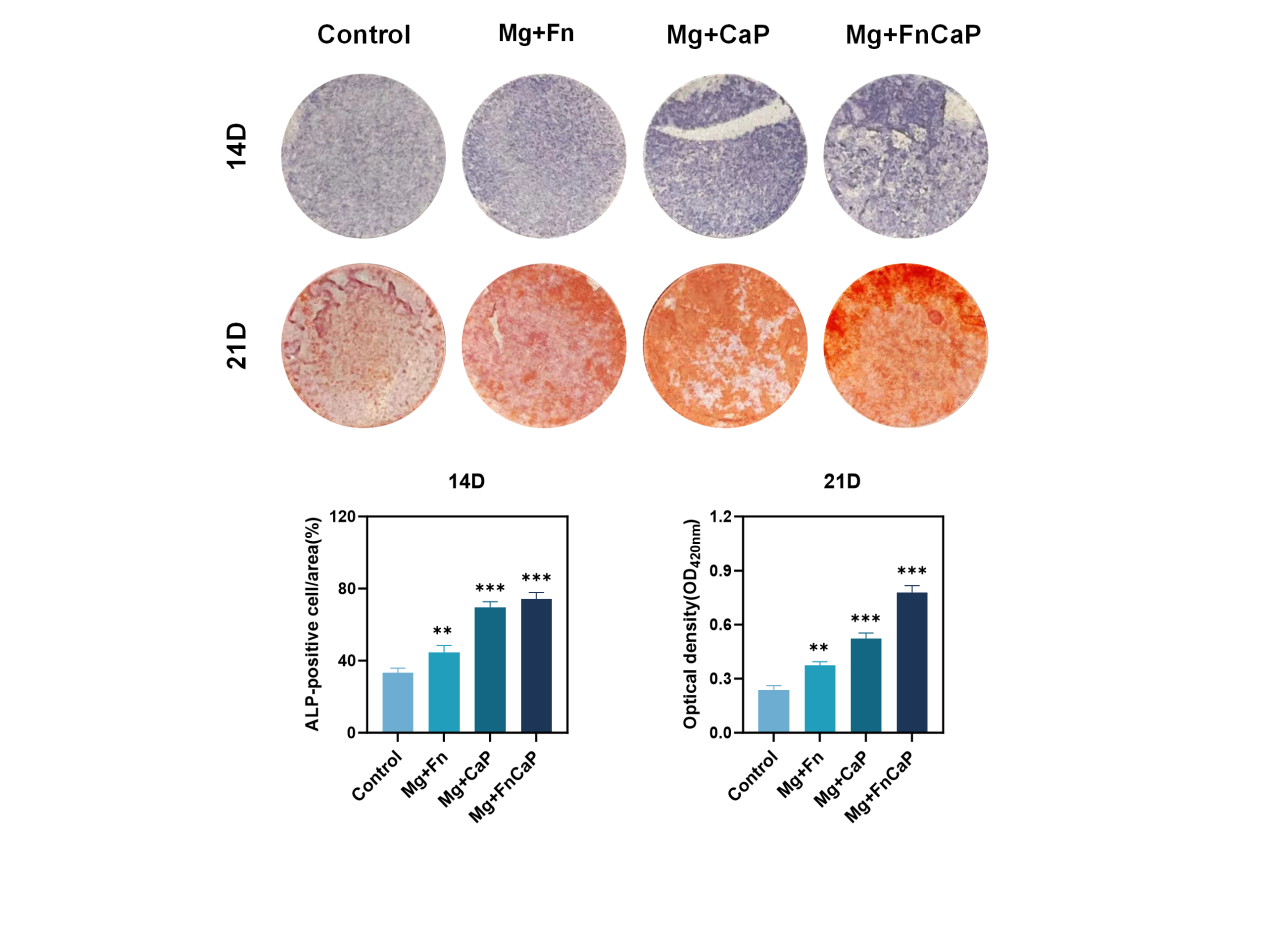


**Figure S17.** ALP and ARS staining of BMSCs cultured in direct contact with Mg alloy surfaces and quantitative results, scale bar = 100 μm. (n = 3). Data shown represent the mean ± SD. Statistical analysis was performed using one-way ANOVA test with a Tukey’s post hoc test. Compared with Control, *p < 0.05, **p < 0.01, ***p < 0.001.


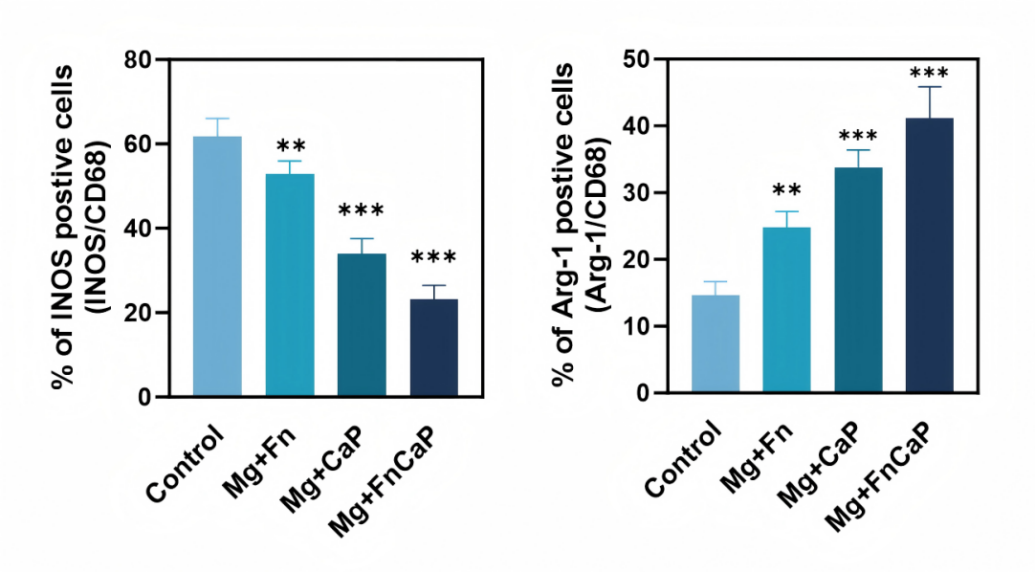


**Figure S18.** Corresponding quantitative data for Figure 7d (n = 5). Data shown represent the mean ± SD. Statistical analysis was performed using one-way ANOVA test with a Tukey’s post hoc test. Compared with Control, *p < 0.05, **p < 0.01, ***p < 0.001.


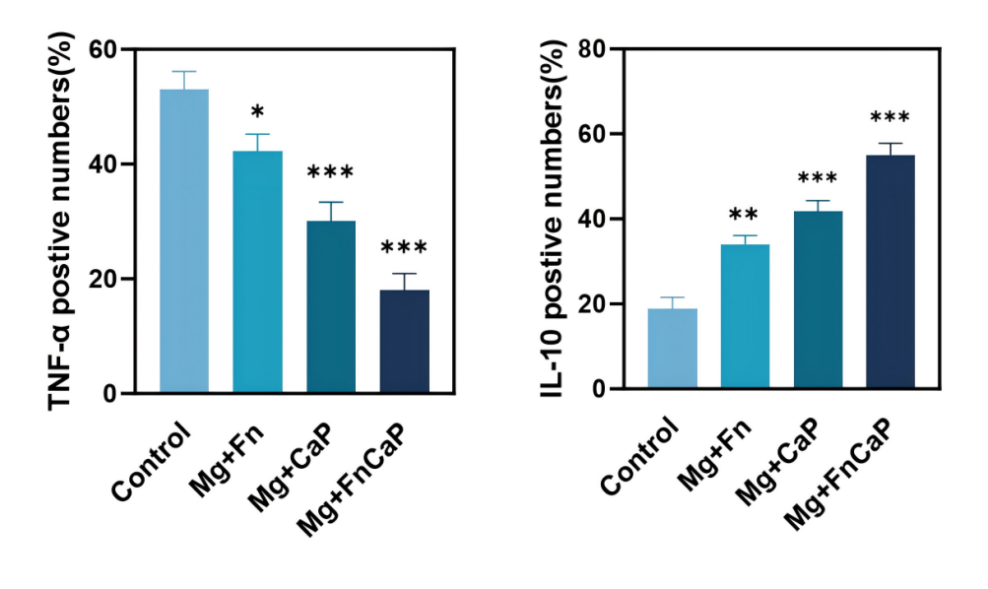


**Figure S19.** Corresponding quantitative data for Figure 7e (n = 5). Data shown represent the mean ± SD. Statistical analysis was performed using one-way ANOVA test with a Tukey’s post hoc test. Compared with Control, *p < 0.05, **p < 0.01, ***p < 0.001.


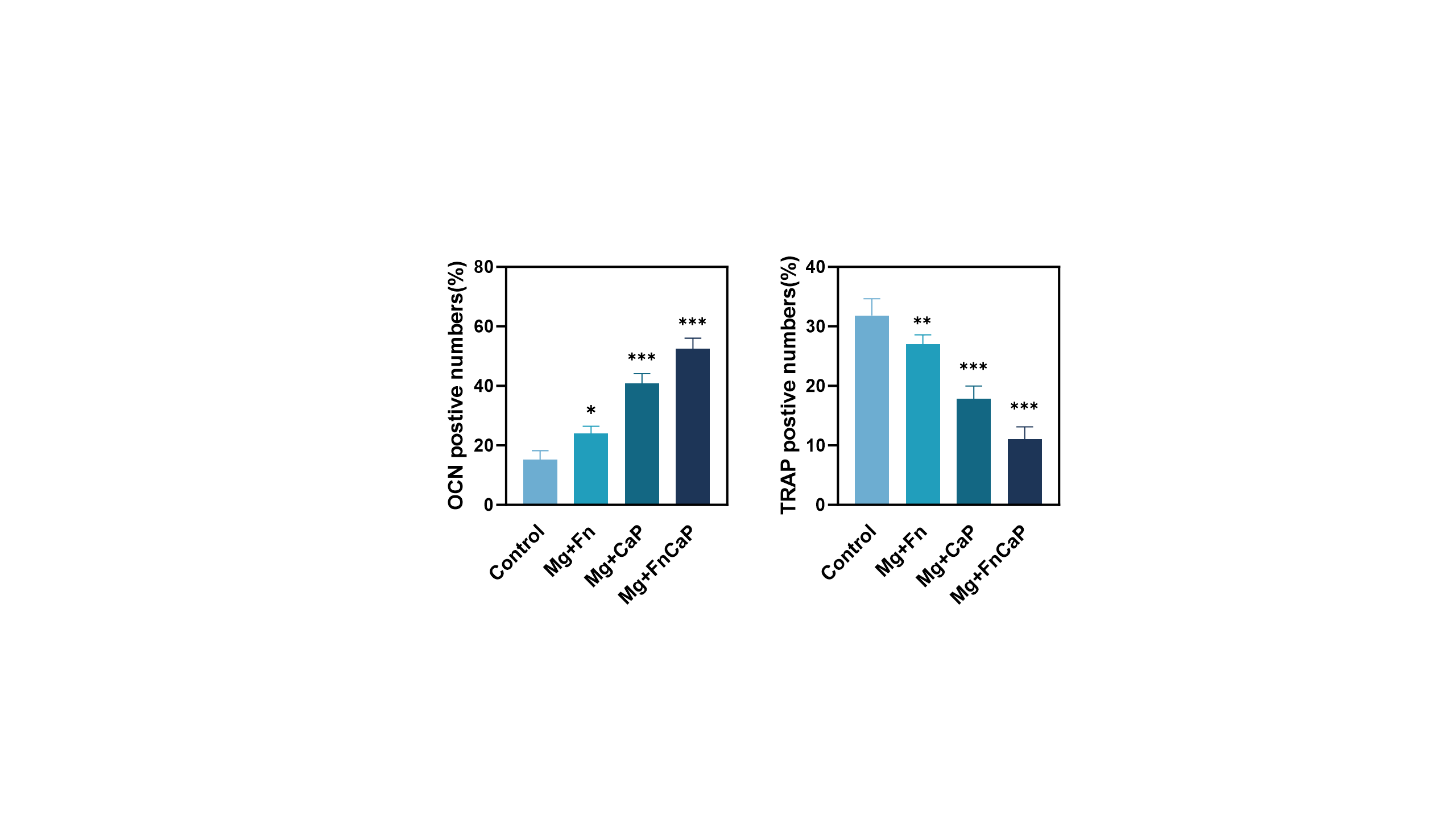


**Figure S20.** Corresponding quantitative data for Figure 8f, h (n = 5). Data shown represent the mean ± SD. Statistical analysis was performed using one-way ANOVA test with a Tukey’s post hoc test. Compared with Control, *p < 0.05, **p < 0.01, ***p < 0.001.


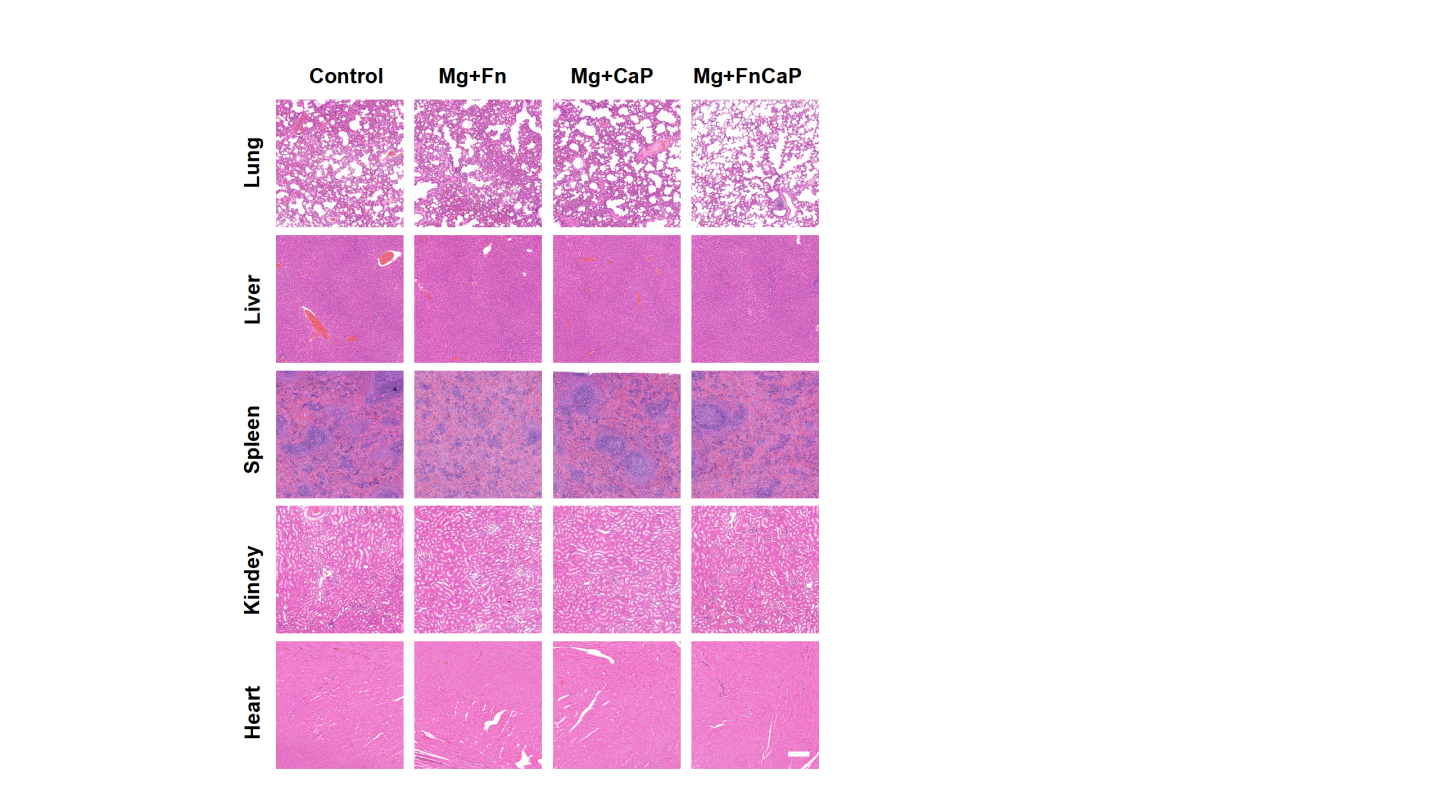


**Figure S21.** H&E staining images of major organs obtained from different groups, scale bar = 100 μm.

Supplementary Table S1. Primers used in the RT-PCR of BMSCs and RAW264.7 cells.

| **Cell** | **Gene** | **Primers Sequence (5‘-3‘)** |
| --- | --- | --- |
| BMSCs | ALP | F:ATGCTCAGGACAGGATCAAA |
|  |  | R: CGGGACATAAGCGAGTTTCT |
|  | Runx2 | R: CGGGACATAAGCGAGTTTCT |
|  |  | F: ATCATTCAGTGACACCACCA |
|  | OPN | F: GAACATGAAATGCTTCTTTCTCAG |
|  |  | R:TCCATGAAGCCACAAACTAAACTA |
|  | OCN | F: GAGGCTCTGAGAAGCATAAA |
|  |  | R: AGGGCAATAAGGTAGTGAA |
|  | GAPDH | F: CCTCTATGACAACACAGT |
|  |  | R: AGCCACCAATCCACACAG |
| RAW264.7 | Arg-1 | F: TGTGTCCAGGCTCCAAATATAG |
|  |  | R: AGCAGGTAGCTGAAGGTCTC |
|  | iNOS | F: CACCAAGCTGAACTTGAGCG |
|  |  | R: CGTGGCTTTGGGCTCCTC |
|  | TNF-α | F: GTTCCCAAATGGCCTCCC |
|  |  | R: GTGCTCCTCACCCACACCG |
|  | IL-10 | F: CCCTTTGCTATGGTGTCCT |
|  |  | R: GTGGCCAGTTTGTTATTTAT |
|  | IL-6 | F: ATAGTCCTTCCTACCCCAATTTCC |
|  |  | R: GATGAATTGGATGGTCTTGGTCC |
|  | IL-1β | F: TGGAGAGTGTGGATCCCAAG |
|  |  | R: GGTGCTGATGTACCAGTTGG |
|  | β-actin | F: GTGACGTTGACATCCGTAAAGA |
|  |  | R: GTAACAGTCCGCCTAGAAGCAC |
|  | P50 | F:GCTGCCAAAGAAGGACACGACA |
|  |  | R:GGCAGGCTATTGCTCATCACAG |
|  | p52 | F:TGCTGATGGCACAGGACGAGAA |
|  |  | R:GTTGATGACGCCGAGGTACTGA |
|  | P65 | F:TCCTGTTCGAGTCTCCATGCAG |
|  |  | R:GGTCTCATAGGTCCTTTTGCGC |
|  | lкBα | F:GCCAGGAATTGCTGAGGCACTT |
|  |  | R:GTCTGCGTCAAGACTGCTACAC |
